# A transcriptome study on *Macrobrachium nipponense* hepatopancreas experimentally challenged with white spot syndrome virus (WSSV)

**DOI:** 10.1371/journal.pone.0200222

**Published:** 2018-07-06

**Authors:** Caiyuan Zhao, Hongtuo Fu, Shengming Sun, Hui Qiao, Wenyi Zhang, Shubo Jin, Sufei Jiang, Yiwei Xiong, Yongsheng Gong

**Affiliations:** 1 Wuxi Fisheries College, Nanjing Agricultural University, Wuxi, PR China; 2 Key Laboratory of Freshwater Fisheries and Germplasm Resources Utilization, Ministry of Agriculture, Freshwater Fisheries Research Center, Chinese Academy of Fishery Sciences, Wuxi, PR China; Kunming University of Science and Technology, CHINA

## Abstract

White spot syndrome virus (WSSV) is one of the most devastating pathogens of cultured shrimp, responsible for massive loss of its commercial products worldwide. The oriental river prawn *Macrobrachium nipponense* is an economically important species that is widely farmed in China and adult prawns can be infected by WSSV. However, the molecular mechanisms of the host pathogen interaction remain unknown. There is an urgent need to learn the host pathogen interaction between *M*. *nipponense* and WSSV which will be able to offer a solution in controlling the spread of WSSV. Next Generation Sequencing (NGS) was used in this study to determin the transcriptome differences by the comparison of control and WSSV-challenged moribund samples, control and WSSV-challenged survived samples of hepatopancreas in *M*. *nipponense*. A total of 64,049 predicted unigenes were obtained and classified into 63 functional groups. Approximately, 4,311 differential expression genes were identified with 3,308 genes were up-regulated when comparing the survived samples with the control. In the comparison of moribund samples with control, 1,960 differential expression genes were identified with 764 genes were up-regulated. In the contrast of two comparison libraries, 300 mutual DEGs with 95 up-regulated genes and 205 down-regulated genes. All the DEGs were performed GO and KEGG analysis, overall a total of 85 immune-related genes were obtained and these gene were groups into 13 functions and 4 KEGG pathways, such as protease inhibitors, heat shock proteins, oxidative stress, pathogen recognition immune receptors, PI3K/AKT/mTOR pathway, MAPK signaling pathway and Ubiquitin Proteasome Pathway. Ten genes that valuable in immune responses against WSSV were selected from those DEGs to furture discuss the response of host to WSSV. Results from this study contribute to a better understanding of the immune response of *M*. *nipponense* to WSSV, provide information for identifying novel genes in the absence of genome of *M*. *nipponense*. Furthermore, large number of transcripts obtained from this study could provide a strong basis for future genomic research on *M*. *nipponense*.

## Introduction

White spot syndrome disease is one of the most destructive viral disease in global shrimp aquaculture, causing considerable economic losses every year and has been estimated at more US$8 billion since 2000 [1–9]. The disease is caused by white spot syndrome virus (WSSV), a double-stranded DNA virus in the genus *Whispovirus*, family *Nimaviridae* [10]. Since its first appearance in Taiwan in 1992, the virus has quickly spread and affected many aquaculture areas worldwide [11, 12]. Almost all decapod crustaceans, including shrimps, crabs, lobsters and crayfish, are considered susceptible to this virus [13, 14]. The oriental river prawn *Macrobrachium nipponense* could be effectively infected by WSSV through oral administration and intramuscular injection [15, 16].

The Oriental river prawn, *Macrobrachium nipponense* is one of the most important economic species that is farmed widely in China [17, 18], with annual yields exceeding 265 061 metric tonnes [19]. Compared with penaeid shrimps, freshwater prawns, especially *M*. *nipponense*, were generally considered to be less prone to disease in culture [16, 20]. *Macrobrachium nipponense*, like other crustaceans, lack an acquired immune system and rely totally on the innate defense system to resist pathogen invasion [21]. The hepatopancreas of crustaceans is the main immune organ, playing an important role in health, growth and survival and has been used as a monitor organ for the overall assessment the health [22, 23]. *Macrobrachium nipponense* can be infected by WSSV and the natural WSSV prevalence level of *M*. *nipponense* was about 8.3% [15, 24]. In our present study, *M*. *nipponense* has a more effective immune reponse to WSSV infection than *L*. *vannamei* and the hepatopancreas in *M*. *nipponense* can be affected by WSSV easily [16]. In addition, *M*. *nipponense* can normally survive with carrying WSSV. The survived *M*. *nipponense* with WSSV reveals that the host conducted a successful and efficient immune responses to against WSSV infection. While, the moribund *M*. *nipponense* due to WSSV infection reveals the host have made a ultimate immune responses to against WSSV. Elucidation of the immune mechanism of WSSV infection in *M*. *nipponense* will be helpful to other crustaceans aquaculture.

In recent years, attempts have been conducted to investigate the effects of WSSV infection on shrimp transcriptome using cDNA microarray, the suppression subtractive hybridization (SSH), expressed sequence tag analysis (EST) and so on [25–27]. However, microarrays and subtractive hybridization methods are impeded by background and cross-hybridization problems, and only the relative abundance of transcripts is measured [28, 29]. The EST sequencing technique is laborious, and it has limitations in sampling the depth of the transcriptome, which could possibly loss the detection of transcripts with low abundance [30]. The next generation sequencing (NGS), a far superior technology, was introduced in 2004 [31, 32]. The expressed sequences produced using NGS technologies are usually 10- to100-fold greater than the number identified by traditional Sanger sequencing technologies in much shorter times [33, 34]. This platform provides an efficient way to rapidly generate large amounts of data with low cost [35, 36]. As a powerful method, comparative transcriptome analysis has been used to discover the molecular basis underlying specific biological events, such as immune processes during infection [37, 38]. However, no information is available on the gene expression profiles of *M*. *nipponense* with a WSSV infection.

In the present study, we applied the next generation sequencing and bioinformatics techniques for analyzing the transcriptome differences from the hepatopancreas of *M*. *nipponense* experimentally infection with WSSV among the survived, moribund and normal control prawns without previously annotated genomes as references. It will demonstrate some immune molecular mechanisms involved in WSSV infection, identify candidate immune-related genes against WSSV infection, and construct possible strategies to prevent the spread of WSSV in aquaculture of other crustaceans.

## Materials and methods

### Ethics statement

The experimental prawns were obtained from a wild population in the Lake Tai, Wuxi city, Jiangsu Province, China, where is not privately-owned or protected. And we purchased prawns from a fisherman lived near the Lake Tai. The object study field did not involve any endangered or protected species. Prawn care and experiments were carried out according to the Care and Use of Agricultural Animals in Agricultural Research and Teaching and approved by the Science and Technology Bureau of China. Approval from the Department of Wildlife Administration was not required for the experiments performed in this paper. All experiments in this paper were produced with permits obtained from the Government of the People’s Republic of China and granted by the Animal Experimentation Ethics Committee of Chinese Academy of Sciences.

### *M*. *nipponense* preparation and WSSV infection

Oriental river prawns, *M*. *nipponense* (body weight 4.56±1.24 g) were purchased from a wild population in Lake Tai, Wuxi city, Jiangsu Province, China. The prawns were acclimatized for 7 days to being challenged by WSSV in a recirculating-water aquarium system filled with aerated freshwater (25±1°C) and fed with paludina (freshwater snails with an operculum) twice daily. The prawns were randomly sampled tested by two-step PCR to ensure they were free of WSSV. The prawns were cultured in two group (experimental and control group). Each group was consisted of 20 prawns and the prawns were stocked in 50 L tank. The experimental group were intramuscularly injected with 20 μl filtered supernatant obtained from WSSV infected *Litopenaeus vannamei* (10^6.8^copies ml^−1^, identified by qRT-PCR). Similarly, the control group were injected with same volume of phosphate-buffered saline. After WSSV infection, three to five hepatopancreas tissues of moribund prawns (prawns that can not swin at all and can only shake their pleopods slightly) at the third day and survived prawns at the seventh day were dissected and immediately frozen in liquid nitrogen before storage at -80°C until RNA extraction. Also, three to five hepatopancreas tissues of control prawns were dissected and immediately frozen in liquid nitrogen before storage at -80°C until RNA extraction. Our previous studies working on LD_50_ of WSSV in *M*. *nipponens* showed the right time of dissecting tissues [16]. Three biological replicates were performed, and the equal molar amount of RNA was pooled for transcriptome sequencing. The hepatopancreas tissues from control prawns were also collected for RNA extraction. Three biological replicates were performed, and the equal molar amount of RNA was pooled for transcriptome sequencing.

### Total RNA extraction and Illumina sequencing (cDNA library construction, and sequencing)

Total RNA was isolated from each samples using the mir*Vana*^*TM*^ miRNA Isolation Kit (Ambion, Life Technologies) following the manufacturer’s protocol. RNA degradation and contamination was evaluated by 1% agarose gel electrophoresis to confirm the quality of RNA. The purity of RNA was assessed with a Nanodrop 2000 (NanoDrop Technologies, Maine, USA). RNA integrity was assessed using the Agilent 2100 Bioanalyzer (Agilent Technologies, Santa Clara, CA, USA). The samples with RNA Integrity Number (RIN) ≥ 7 were subjected to the subsequent analysis, including mRNA purification, cDNA library construction and transcriptome sequencing. Approximately 5 μg of total RNA representing each group was used to construct a cDNA library following the protocols of the Illumina TruSeq Stranded mRNA LTSample Prep Kit (Illumina, San Diego, CA, USA), including the purification of mRNA from 5 μg of total RNA using Sera-mag Magnetic Oligo (dT) Beads (Illumina), fragmentation of mRNA, synthesis of the first-strand cDNA with a random hexamer primer and then synthesis of second-strand cDNA, cDNA end repair and adenylation at the 3’ end, adapter ligation and cDNA fragment enrichment, purification of cDNA products. After necessary quantification and qualification, the library was sequenced suing an Illumina HiSeq^TM^ 2500 instrument with 125 bp paired-end (PE) reads for moribund prawns, survived prawns and control prawns.

### De novo assembly and gene annotation

After sequencing, the raw reads were filtered to generate clean reads. The clean reads were obtained by removing adaptor sequence, reads containing ploy-N and low quality sequences (with a quality score less than 20) [32, 39]. The remaining clean reads were assembled using Trinity software with default parameters [40, 41]. Generally, three steps were performed, including Inchworm, Chrysalis and Butterfly [42]. In the first step, clean reads were operated by Inchworm to form longer fragments, called contigs. Then, contigs were identified connection by Chrysalis to obtain unigens, which could not be extended on either end. Uingens resulted in de Bruijn graphs. Finally, transcripts were obtained by using Butterfly to process the de Bruijn graphs [43].

After de novo assembly of clean reads was finished, assembled contigs were annotated with sequences available in the NCBI database, using the BLASRx and BLASTn algorithms [44]. The unigenes were aligned by a homology search (BLASTx) (Mount, 2007) with an E-value cut-off of 10^−5^ against NCBI non-redundant (Nr) database, Swissprot [45], Clusters of Orthologous Groups for Eukaryotic Complete Genomes database (KOG) and Kyoto Encyclopedia of Genea and Genome (KEGG) database [46]. Then, the sequence direction and protein-coding-region prediction (CDS) of the unigenes were determined using the best alignment results. In addition, the Blast2GO software [47] was used to obtained the Gene Ontology (GO) [48] annotations of the uniquely assembled transcripts. KEGG annotation was conducted using KAAS software. For those unigenes without annotation, the coding sequence and direction were predicted using an ESTscan, with BLAST predicted coding sequence data [49].

### Identification of differentially expressed unigenes

In this study, the FRKM (Fragments Per Kb per Million reads) was used as an unit of measurement to estimate the transcript expression levels of the unigenes. The FPKM method can eliminate the influence of gene length and sequencing on the calculation of gene expression. Therefore, the calculated gene expression can be used directly to compare the differences of gene expression between samples. Furthermore, we also used FDR (false discovery rate) to correct for the P-value [50]. Genes with FDR less than 0.001 and a FPKM ratio larger than 2 or smaller than 0.5 were considered as DEGs (differential expression genes) in a given library. Using this method, DEGs were identified between samples through a comparative analysis of the above data. Furthermore, all differentially expressed genes were mapped to terms in GO and KEGG database, and looked for significantly (P-value ≤ 0.05) enriched GO and KEGG terms in DEGs compared to the transcriptome background [51].

### Quantitative real-time PCR validation

The validation of Illumina sequencing results involved the ten selected differentially expressed unigenes of *M*. *nipponense* (TSPAN8, UDP-N-acetylglucosamine—dolichyl-phosphate N-acetylglucosaminephosphotransferase, Gag-Pol polyprotein, arylsulfatase B, MAP kinase-activated protein kinase 2, pyruvate dehydrogenase (acetyl-transferring) kinase, integrin-linked protein kinase, acid phosphatase, ERI1 exoribonuclease 3 and zinc finger MYM-type protein 1) for quantitative real-time reverse transcription polymerase chain reaction (qRT-PCR) analysis. The ten genes were selected from the common part of two libraries, the library of comparison between control and moribund samples and the library of comparison between control and survived samples. The tissues used for qRT-PCR were same as the samples used in transcriptome sequencing. Three hepatopancreas tissues of one group was uniform mixed as a mixed sample. Total RNA of samples was extracted using a high-purity total RNA Rapid Extraction Kit (BioTeke, Beijing, China). The first strand of cDNA was synthesized using EasyScript One-Step gDNA Removal and cDNA Synthesis SuperMix (TransGen Biotech, China) according to the manufacturer’s instructions. Optimized qRT-PCR reactions were performed following the manufacturer’s instructions (SYBR Premix Ex Taq, Takara Bio, Japan.) on a real-time thermal cycler (Bio-Rad, USA), using β-actin as a reference gene. Primers for qRT-PCR were carefully designed using Primer Premier 3 software and listed in Table 1. The qRT-PCR program conducted as showed in Zhao et al [16]. Each sample was repeated in triplicate and 2^-ΔΔCT^ methods were used to calculate the expression level of the ten selected genes. The value of log _2_-fold changes was used for the graphing.

**Table 1:** Primer sequences of genes used for quantitative real-time PCR.

Name	Sequence
TSPAN8-F	5’-ACT-ACT-AAC-TTC-AGC-CTA-TCTAG-3’
TSPAN8-R	5’-ACCGAGTCAAGTAGGGATAGTCA-3’
DPAGT1-F	5’-ACTGGAGGCATTTCTTGTCTGAT-3’
DPAGT1-R	5’-CGCATGTCCTACCTCTTGTATCA-3’
Gag-pol-F	5’-AGTATAATTTCTGGTGCGCGGTA-3’
Gag-pol-R	5’-ATCAGACAAGAAATGCCTCCAGT-3’
ARSB-F	5’-GCCTCTGTTCAATGAGGTTTGAC-3’
ARSB-R	5’-CTGTGTGAAGACGAGAAGAAAGC-3’
MKK2-F	5’-CTGACCCAGAAGAAAGGATGACA-3’
MKK2-R	5’-AGTGTATCGTAATCCACACGCAT-3’
PDK-F	5’-GCTACAAAACGATGATGGTGCAT-3’
PDK-R	5’-CCAAAACTCCACACATCTGATCG-3’
ILK-F	5’-GGGGCAGGATTGTGACTCTTAT-3’
ILK-R	5’-ATCCCAGCCTCTTTGTTTGACA-3’
AP-F	5’-TGGCCAAACACTTAAAGAGCGTA-3’
AP-R	5’-GGTCCTGTACGTGGAAGAACTC-3’
ERI3-F	5’-CAACGATCCAGGCAGTAAAAAGG-3’
ERI3-R	5’-GGAAACTGGTGAAACTCAAAGCA-3’
ZMYM1-F	5’-ATTTCCCGCACTCGTGATCTATT-3’
ZMYM1-R	5’-CCCTGCCACCAGTATTCATTTTC-3’
GST-F	5’-AATGTGGACCAGCCCCAAAG-3’
GST-R	5’-TGCTTTCTTGCAATGTACCGC-3’
Cu/ZnSOD 4 -F	5’-AGTTTCAGCCGTCTGTTCG-3’
Cu/ZnSOD 4 -R	5’-CACAGTGCTTACATCACCCTTA-3’
HSP21-F	5’-GCCTCTGCTCAAGCTAGTGT-3’
HSP21-R	5’-TGGTGGAACCTCCAACAAGG-3’
IRF-F	5’-CCTCTGTGTGAAGACGAGAAGAA-3’
IRF-R	5’-CAAGTGCCCAGTATCTGAAAAGC-3’
ERK-F	5’-CGTTTTCTTTCGCATCTCATCCA-3’
ERK-R	5’-GACGGGGATGAAAATAATGCGAG-3’
MKK4-F	5’-TCCGTTGCTACAAAACGATGATG-3’
MKK4-R	5’-CCGTGTCTTGTGTGAGTTCATTC-3’
JNK-F	5’-ATTATGTAGAAAACCGTCCCCGT-3’
JNK-R	5’-CCTTCGCTCTGGATCAATCACTA-3’
IAP-F	5’-AAAGCCTTGAATTTCCCTGAAGC-3’
IAP-R	5’-AATTAGTTGTGGAGGGTGAAGCA-3’
Ferritin-F	5’-TCAGAAATGCAGAAAAAGCTGGG-3’
Ferritin-R	5’-GTGTTCTAAGTCCTCCCTTCCAA-3’
β-actin-F	5’-TGGACGTGTGGAGAGTGGCATC-3’
β-actin-R	5’-ATCGCCTGGAACTGCCTCAGTC-3’

### The responses of immune-related genes in *M*. *nipponense* to WSSV challenge

*M*. *nipponense* (body weight 4.56±1.24 g, n = 50) were received an intramuscular injection with WSSV inoculum (20 μl). The concentration of WSSV injected was 40% of the LD_50_ for *M*. *nipponense* [16]. Hepatopancreas were collected at 0, 12, 24, 48, 72, 96 h post-inoculation (hpi) with WSSV. At each time point, three individuals were selected randomly. Total RNA extraction and cDNA synthesis were performed as described above. The immune-related genes expression pattern in hepatopancreas were detected by qRT-PCR. The β-actin gene was used as an endogenous control. All samples were tested in triplicate.

## Results

### Transcriptome sequencing and de novo assembly

**Fig 1:**
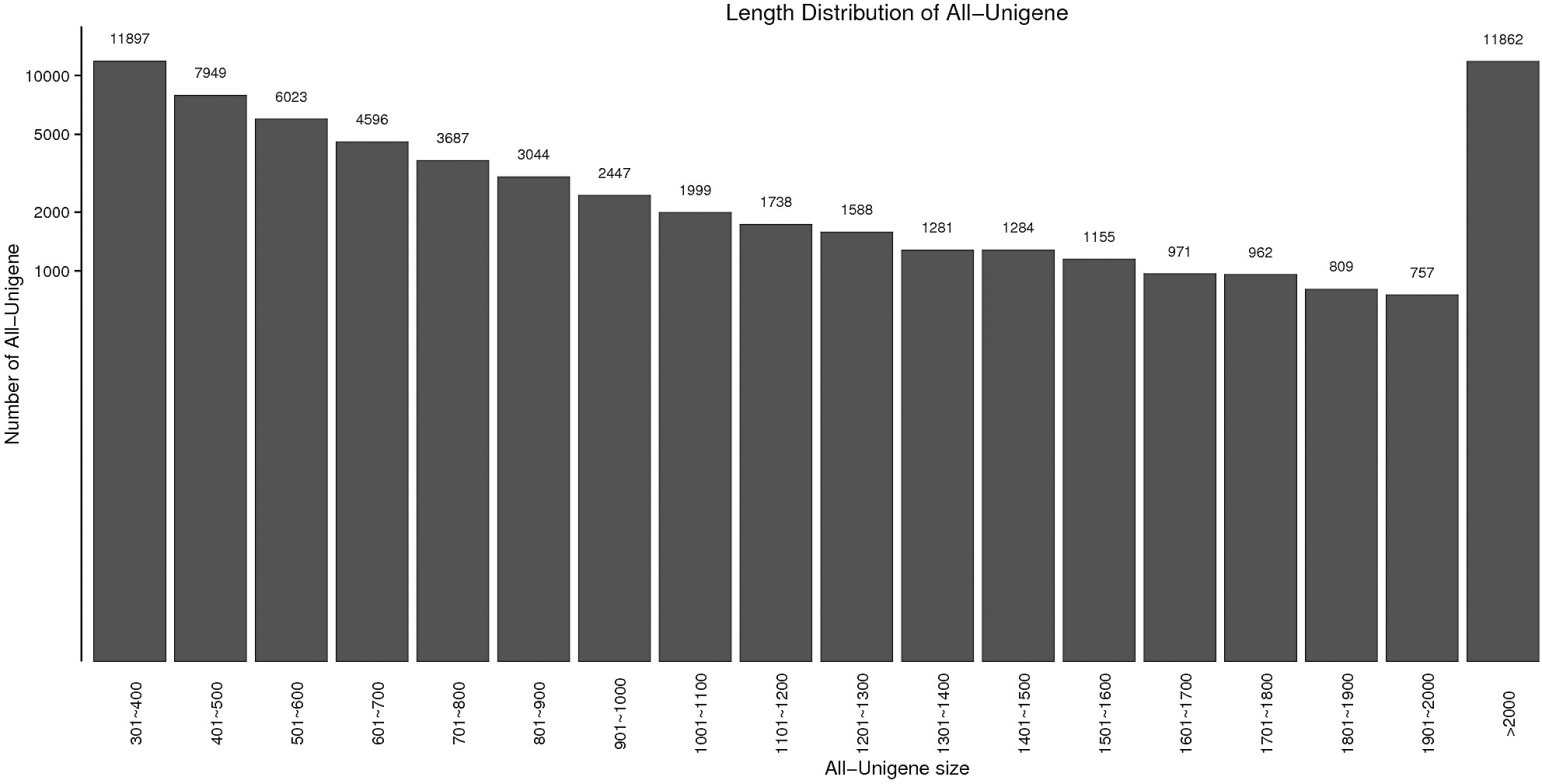
Length distribution of assembled transcripts and unigenes.

The mission to identify immune-related genes which are vital for *M*. *nipponense* defence against WSSV infection involved preparing three cDNA libraries using pooled mRNAs obtained from the hepatopancreases of moribund prawns, survived prawns and control prawns. The Illumina Hiseq2500^TM^ sequencing platform was used to perform high-throughput sequencing. Then, the sequencing data were subjected to de novo assembly using the Trinity program. A total of 52,605,014, 51,924,787 and 51,969,106 raw reads were produced in control, moribund and survived samples, respectively (Table 2). And the PCA analysis result was showed in S1 Fig. After the removal of adapter sequence and low-quality reads, a total of 50,948,257, 50,696,076, and 49,870,542 high quality clean reads that represent a total of 7,635,728,511 (7.63 Gb), 7,599,313,025 (7.60 Gb), 7,475,646,918 (7.48 Gb) nucleotides were generated for the control, moribund and surivived samples, respectively (Table 2). The sequencing reads were found to be high quality with the Average Q_30_ value of 93.53% and their percentage of unknown nucleotide (N percentage) of 0%. The GC content of nucletotide was 44.33%, 44.00% and 44.00% in control, moribund and survived groups, respectively (Table 2). All sequencing reads were stored into the Short Read Archive (SRA) of the National Center for Biotechnology Information (NCBI), and available with the accession PRJNA437347 (https://www.ncbi.nlm.nih.gov/bioproject/PRJNA437347). In detail, the accession number SRR6820546, SRR6820539, SRR6820540 for three control samples, SRR6820543, SRR6820544, SRR6820545 for three moribund samples and SRR6820541, SRR6820542, SRR6820538 for survived ones. The size and length distribution of the control and WSSV-infected unigenes are shown in Fig 1. Among them, the 39,621-length unigenes (61.86%) were less than 1,000 bp, and 19,775-length unigenes (30.87%) were less than 500bp.

**Table 2:** Quality of sequencing data.

Sample	Raw reads	Clean reads	Clear base pairs (bp)	Q30 (%)	N(%)	GC(%)
control_sample1	52,584,444	51,011,136	7,645,216,841	93.49%	0.00%	44.00%
control_sample2	52,758,426	51,257,620	7,682,256,321	93.68%	0.00%	44.00%
control_sample3	52,472,172	50,576,016	7,579,712,371	92.82%	0.00%	45.00%
moribund_sample1	50,682,176	50,081,386	7,509,930,143	95.19%	0.00%	44.00%
moribund_sample2	52,553,448	51,084,812	7,656,384,701	93.72%	0.00%	44.00%
moribund_sample3	52,538,736	50,922,030	7,631,624,230	93.46%	0.00%	44.00%
survived_sample1	50,694,464	50,013,362	7,499,387,721	94.81%	0.00%	43.00%
survived_sample2	52,533,610	51,005,096	7,644,309,325	93.61%	0.00%	44.00%
survived_sample3	52,679,244	48,593,168	7,283,243,709	90.95%	0.00%	45.00%

### Functional annotation and classification of *M*. *nipponense* transcriptome sequences

Protein annotation includes Gene Ontology (GO), KOG and protein functional annotations, which may provide some functional and expression information on the protein. To achieve protein identification and gene annotation, before the analysis of DEGs associated with white spot syndrome virus (WSSV) infection, all sequences from 64,049 unigenes (unigenes from the merged group, control, moribund and survived groups) were annotated using BLASTX (E-value < 10^−5^). In total, 21,063 (32.89%) unigenes were annotated. A total of 20,187 (31.52%), 17,578 (27.44%), 15,211 (23.75%), 7,759 (12.11%), 16,573 (25.88%) unigenes had significant matches with sequences in the NR, Swiss-prot, KOG, KEGG and GO databases, respectively (Table 3). In brief, 20,187 (31.52% of the total unigenes) assembled unigenes aligned to the NR protein database, while the remaining 876 (4.16%) did not hit homology in the database. Among the aligned unigenes, 74.06% members showed strong homology with an E-value of less than 10^−30^ with the gene sequence in the database, whereas the remaining 25.94% unigenes had an E-value ranging from 10^−5^ to 10^−30^ (S2 Fig). Next, we also assembled the species distribution of the unigenes by aligned sequences against the NR protein database to learn the sequence conversation of *M*. *nipponense* compared with other species. Over 47% of the total unigenes matched with sequences from seven top-hit species, i.e., Hyalella azteca (4000, 19.81%), Mus musculus (2201, 10.9%), Opisthorchis viverrini (883, 4.73%), Clonorchis sinensis (832, 4.12%), Zootermopsis nevadensis (565, 2.80%), Limulus polyphemus (418, 2.07%), Daphnia magna (403, 2.00%), and Schistosoma mansoni (226, 1.12%), all of which belong to arthropoda (S3 Fig).

**Table 3:** Summary of annotations of all assembled unigenes.

	Number of unigenes	Percentage (%)
Total length of unigenes	84,694,421	
Mean length of unigenes	1,322.34	
N50 of unigenes	2,205	
Total number of unigenes	64,049	100%
Total annotated	21,063	32.89%
NCBI Nr annotated	20,187	31.52%
Swiss-Prot annotated	17,578	27.44%
KOG annotated	15,211	23.75%
KEGG annotated	7,759	12.11%
GO annotated	16,573	25.88%

**Fig 2:**
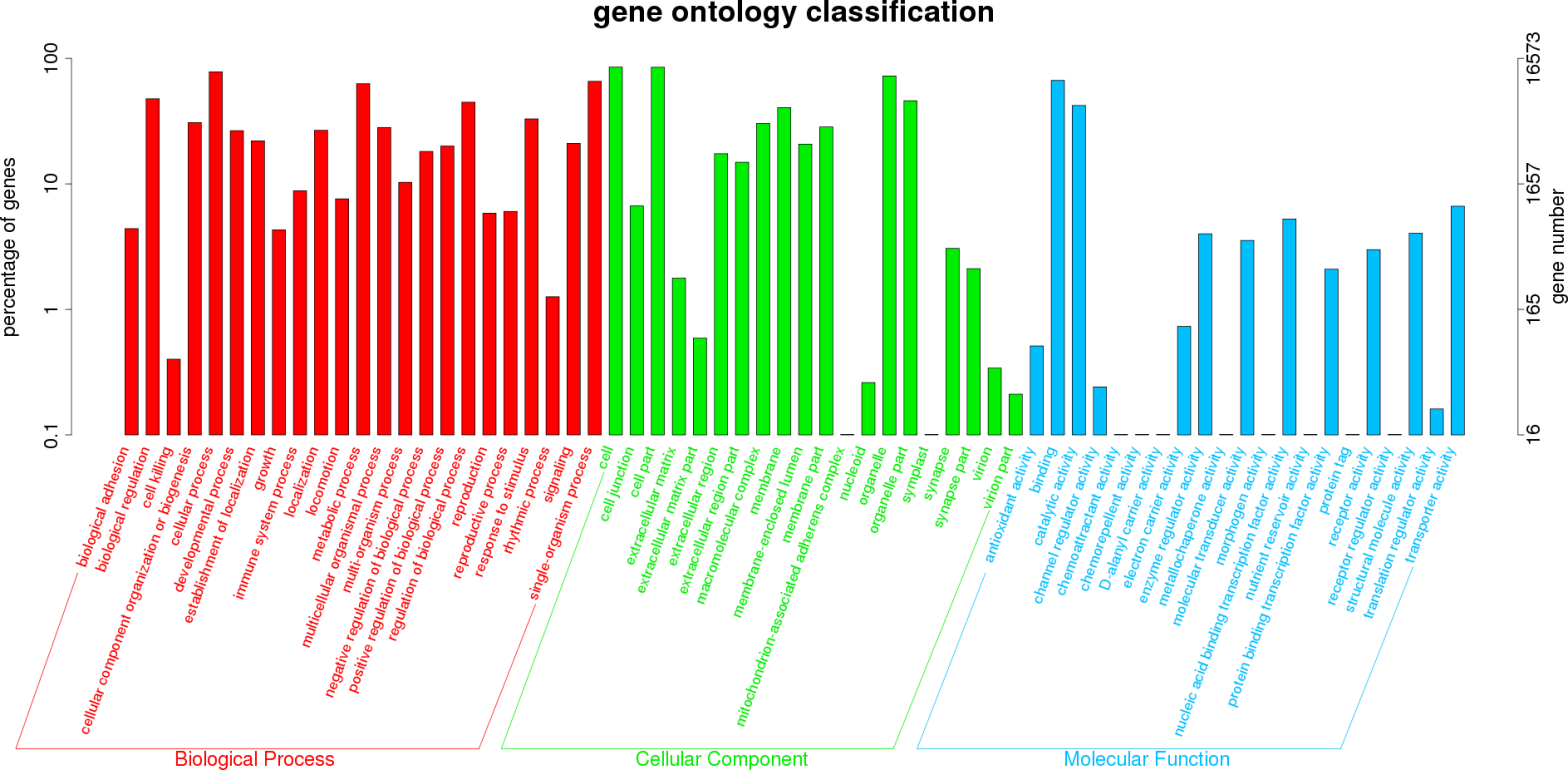
Gene ontology (GO) classification of 16,573 protein annotated unigenes from the three hepatopancreas samples (control, moribund and survived groups). The three main GO categories include biological process (red), cellular component (green), and molecular function (blue). Each bar represents the relative abundance of unigenes classified under each category at level 2.

The Gene Ontology (GO) classification is a unified gene functional classification system that provides a dynamically update controlled vocabulary in a strictly defined gene properties and products in any organism. Sequence homology based on GO classification revealed that 16,573 matched unigenes (25.88% of total unigenes) were divided into three categories, containing 63 functional groups. (Fig 2). We obtained a total of 193,811 GO assignments, where 49.10% comprised biological processes, 38.98% comprised cellular component, and 11.92% comprised molecular function. In biological process category, most unigenes were involved in the “cell process” (20.22%), “single-organism process” (16.99%), and “metabolic process” (16.26%). In the category of cellular component, the most represented were “cell” (22.05%), “cell part” (22.01%), and “organelle part” (18.71%). As for molecular function category, “binding” (17.26%), “catalytic activity” (10.91%), and “transporter activity” (1.72%) were the dominant groups (Fig 2).

The KOG is a database including classified orthologous gene products. Every protein in KOG is assumed to originate from an ancestor protein, therefore the database is developed based on coding proteins with complete genomes and the system evolution relationship of bacteria, algae and eukaryotic creatures. The standard unigenes were aligned to the KOG database to predict and classified their possible roles [32]. KOG classification indicated that 15,211 matched unigenes (23.75% of total unigenes) resulted in 20,187 functional annotiations, and these unigenes were clustered into 25 functional categories (S4 Fig). The largest one was “General function prediction only” (3,657, 17.67%). The second largest was “Signal transduction mechanisms” (2,037, 10.09%), followed by “Posttranslational modification, protein turnover, chaperones” (1,564, 7.74%), “Intracellular trafficking, secretion, and vesicular transport” (926, 4.59%), and “Transcription” (846, 4.19%). The two smallest groups were “Nuclear structure” (81, 0.40%) and “cell motility” (29, 0.14%) (S4 Fig).

In addition, we also used KEGG to identify potential biological pathways involved in the transcriptome of the hepatopancreas of *M*. *nipponense*. A total of 7,759 unigenes (12.11% of total unigenes) were assigned to 376 KEGG pathways into six categories (S5 Fig). KEGG analysis revealed that a great number of genes expressed in *M*. *nipponense* were involved with human disease (9,525, 29.15%), organismal system (7,204, 22.03%), and metabolism (4,878, 14.91%). Of the 9,525 unigenes in the human disease category, 3,677 members (38.60%) were infectious diseases and 2,818 members (29.59%) were cancers. Among these KEGG pathways, the top 50 statistically significant KEGG classification are shown in Table 4. Some important innate immunity-related pathways were predicted in this KEGG database, including lysosome (337, 1.34%), PI3K-Akt signaling pathway (310, 4.00%), Epstein-Barr virus infection (284, 3.66%), HTLV-I infection (279, 3.60%), focal adhesion (269, 3.47%), apoptosis (248, 3.20%), Ras signaling pathway (234, 3.02%), MAPK signaling pathway (225, 2.90%), cAMP signaling pathway (203, 2.62%), NOD-like receptor signaling pathway (177, 2.28%), mTOR signaling pathway (174, 2.24%).

**Table 4:** The top 50 statistically signifcant KEGG classifcations. The rank of a pathway is based on the number of all unigenes that classified into this pathway.

No.	Pathway ID	Pathway definition	Number of unigenes
1	ko05200	Pathways in cancer	381(2.90%)
2	ko04144	Endocytosis	347(2.65%)
3	ko04142	Lysosome	337(2.58%)
4	ko05165	Human papillomavirus infection	326(2.50%)
5	ko04141	Protein processing in endoplasmic reticulum	324(2.49%)
6	ko05016	Huntington's disease	324(2.50%)
7	ko05010	Alzheimer's disease	312(2.42%)
8	ko04151	PI3K-Akt signaling pathway	310(2.41%)
9	ko05205	Proteoglycans in cancer	308(2.40%)
10	ko05169	Epstein-Barr virus infection	284(2.23%)
11	ko05166	HTLV-I infection	279(2.20%)
12	ko03040	Spliceosome	278(2.20%)
13	ko04145	Phagosome	277(2.20%)
14	ko03010	Ribosome	275(2.19%)
15	ko03013	RNA transport	272(2.18%)
16	ko04510	Focal adhesion	269(2.16%)
17	ko05203	Viral carcinogenesis	267(2.15%)
18	ko04810	Regulation of actin cytoskeleton	257(2.09%)
19	ko04932	Non-alcoholic fatty liver disease (NAFLD)	252(2.06%)
20	ko04210	Apoptosis	248(2.03%)
21	ko04015	Rap1 signaling pathway	244(2.01%)
22	ko05012	Parkinson's disease	242(2.00%)
23	ko04530	Tight junction	241(2.00%)
24	ko00190	Oxidative phosphorylation	238(1.99%)
25	ko04014	Ras signaling pathway	234(1.96%)
26	ko04010	MAPK signaling pathway	225(1.90%)
27	ko00230	Purine metabolism	222(1.89%)
28	ko05164	Influenza A	219(1.87%)
29	ko04140	Autophagy—animal	216(1.86%)
30	ko05167	Kaposi's sarcoma-associated herpesvirus infection	209(1.81%)
31	ko01200	Carbon metabolism	208(1.81%)
32	ko05418	Fluid shear stress and atherosclerosis	204(1.79%)
33	ko04013	MAPK signaling pathway—fly	204(1.80%)
34	ko04024	cAMP signaling pathway	203(1.80%)
35	ko05168	Herpes simplex infection	201(1.79%)
36	ko04218	Cellular senescence	198(1.77%)
37	ko04120	Ubiquitin mediated proteolysis	195(1.76%)
38	ko04921	Oxytocin signaling pathway	191(1.74%)
39	ko05206	MicroRNAs in cancer	184(1.69%)
40	ko04910	Insulin signaling pathway	183(1.69%)
41	ko05152	Tuberculosis	181(1.68%)
42	ko04062	Chemokine signaling pathway	180(1.68%)
43	ko04621	NOD-like receptor signaling pathway	177(1.67%)
44	ko04360	Axon guidance	176(1.67%)
45	ko04022	cGMP—PKG signaling pathway	176(1.68%)
46	ko04150	mTOR signaling pathway	174(1.67%)
47	ko04217	Necroptosis	174(1.68%)
48	ko04919	Thyroid hormone signaling pathway	164(1.61%)
49	ko05161	Hepatitis B	162(1.60%)
50	ko04020	Calcium signaling pathway	160(1.59%)

Based on the NR, Swiss-prot and KEGG database, 21,134 and 12,629 CDSs were predicted by BLASTX and ESTScan, respectively (S6 Fig). Among these CDSs, 389 were over 2,000 bp and 10,455 were over 500 bp. Unigenes not identified as coding sequences were probably etihter non-coding RNAs or were too short to reach the criterion of CDS prediction, and need to verified in the future study.

### Identification and functional characterization of differentally expressed genes (DEGs)

**Fig 3:**
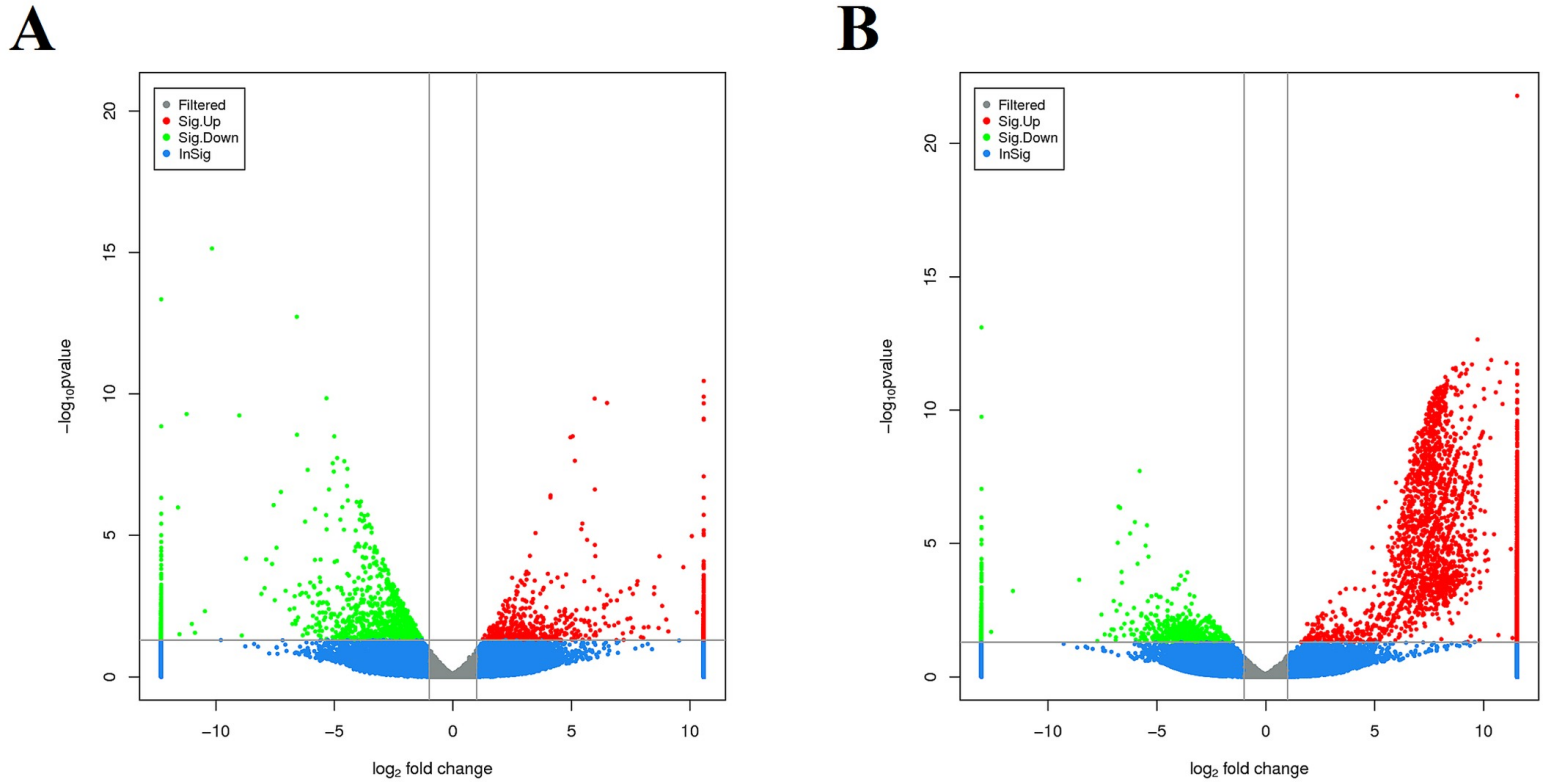
**Digital gene expression between moribund samples and control (A), digital gene expression between survived samples and control (B).** Each unigene was presented as a point. The *x*- and *y*-axis are the log_2_-fold change and the log_10_ P-value of the normalized expression level (FPKM) of gene between the two compared groups. Red and green points indicate aberrantly change at the absolute value of log_2_ (FPKM ratio in two compared groups) ≥ 1 and FDR ≤ 0.001. Red points represent up-regulated genes. Green points represent down-regulated genes. Blue points represent insignificant differentially expressed genes.

**Fig 4:**
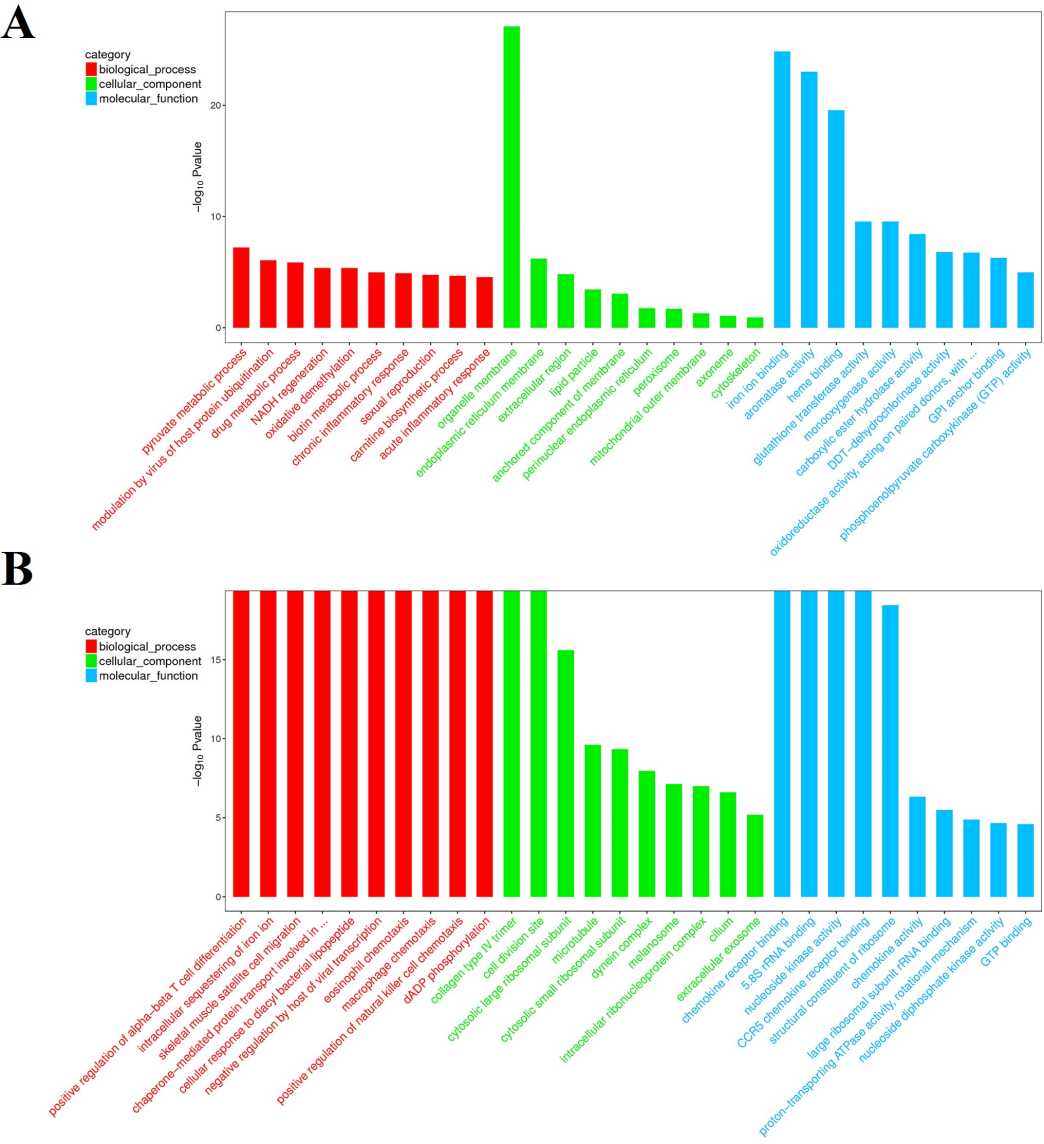
The top 10 GO enrichment terms of DEGs in each category at level 2. The three GO categories include biological process (red), cellular component (green), and molecular function (blue). A means the enrichment GO terms of DEGs from the comparison between moribund samples and control. B means the enrichment GO terms of DEGs from the comparison between survived samples and control. The *x*- and *y*-axis are the GO terms and the log_10_ P-value of corresponding GO terms.

In order to identify the DEGs involved in WSSV infection in the hepatopancreas of *M*. *nipponense*, a comparison of the relative relative transcript abundance in each unigenes was performed using the FPKM algorithm (fragments Per kb per Million reads) with a threshold absolute value of log_2_ fold-change ≥ 1 and FDR ≤0.001. In the comparison of moribund samples and control, a total of 1,960 genes were found to be aberrantly expressed, with 764 significantly up-regulated and 1,196 significantly down-regulated (Fig 3A). In brief, among these 1,960 DEGs, 1,329 genes were expressed in both control and moribund groups, including 443 significantly up-regulated genes and 886 significantly down-regulated genes. Moreover, 321 genes were only expressed in the moribund samples after WSSV challenge. To confirm the biological function of DEGs, GO functional and KEGG pathway enrichment analysis for the DEGs were performed. GO functional enrichment analysis results from the DEGs of comparison between moribund samples and control indicated that 438 of the 1,960 DEGs had a GO ID and categorized into 1,481 functional groups in three categories: biological process, cellular component and molecular function. The top 10 terms of each category were showed in Fig 4A. Most of the GO terms were involved in the molecular function, such as “iron ion binding”, “aromatase activity” and “heme binding”, which were over-represented. The enrichment analysis results also reveal that GO terms “pyruvate metabolic process” and “modulation by virus of host protein ubiquitination” were the most represented terms in the biological process category. Of all the terms in three categories, the GO term “organelle membrane” which belong to the cellular component category were the most significantly over-represented (P-value of 8.24E-28). These pyruvate metabolic and modulation by virus of host protein ubiquitination process may act important roles in the WSSV reponse in the hepatopancreas of *M*. *nipponense* and most the reponses occurred in the “organelle membrane”. To further investigate the biochemical pathways of DEGs, all of the DEGs from comparison between moribund samples and control were mapped in the KEGG database and compared with the whole transcriptome background to search for genes refer to the innate immune response or signaling pathway. Of the 1,960 DEGs, 244 genes had a KO ID and were associated with 230 pathways (67 up-regulated genes categories into 106 pathways, and 177 down-regulated genes categories into 201 pathways). KEGG pathway enrichment analysis results showed that 44 pathways were obviously changed (P-value < 0.05) in the moribund samples compared with the control samples and the top 20 enriched pathways were showed in Table 5, with genes involved in “Metabolism of xenobiotics by cytochrome P450”, “Retinol metabolism”, “Ascorbate and aldarate metabolism” and “Glutathione metabolism” being the most significantly enriched. And 9 of the 1,960 DEGs were enriched on Peroxisome (Table 5).

**Table 5:** Top 20 differentially expressed pathways of two comparison libraries.

**Top 20 differentially expressed pathways between the moribund samples and control.**				
**NO.**	**Pathway ID**	**Pathway**	**Number of DEGs**	**P-value**
1	ko00980	Metabolism of xenobiotics by cytochrome P450	29(1.48%)	2.04E-25
2	ko05204	Chemical carcinogenesis	29(1.48%)	3.90E-24
3	ko00982	Drug metabolism—cytochrome P450	25(1.28%)	4.87E-22
4	ko00981	Insect hormone biosynthesis	16(0.82%)	4.55E-16
5	ko00040	Pentose and glucuronate interconversions	19(0.97%)	3.00E-14
6	ko00830	Retinol metabolism	18(0.92%)	8.91E-14
7	ko00983	Drug metabolism—other enzymes	18(0.92%)	8.45E-13
8	ko00140	Steroid hormone biosynthesis	14(0.71%)	8.22E-12
9	ko00053	Ascorbate and aldarate metabolism	12(0.61%)	6.38E-10
10	ko00480	Glutathione metabolism	16(0.82%)	2.38E-09
11	ko00860	Porphyrin and chlorophyll metabolism	14(0.71%)	2.92E-09
12	ko00590	Arachidonic acid metabolism	9(0.46%)	4.67E-06
13	ko01524	Platinum drug resistance	11(0.56%)	6.45E-06
14	ko04977	Vitamin digestion and absorption	7(0.36%)	7.62E-06
15	ko00780	Biotin metabolism	3(0.15%)	3.10E-05
16	ko00100	Steroid biosynthesis	4(0.20%)	0.000181
17	ko01220	Degradation of aromatic compounds	3(0.15%)	0.000991
18	ko05033	Nicotine addiction	2(0.10%)	0.00153
19	ko00604	Glycosphingolipid biosynthesis—ganglio series	3(0.15%)	0.003107
20	ko04146	Peroxisome	9(0.16%)	0.004405
**Top 20 differentially expressed pathways between the survived samples and control.**				
**NO.**	**Pathway ID**	**Pathway**	**Number of DEGs**	**P-value**
1	ko03010	Ribosome	104(2.41%)	5.91E-18
2	ko05130	Pathogenic Escherichia coli infection	43(1.00%)	3.60E-07
3	ko04145	Phagosome	78(1.80%)	5.92E-07
4	ko00710	Carbon fixation in photosynthetic organisms	19(0.44%)	6.01E-05
5	ko04022	cGMP—PKG signaling pathway	49(1.14%)	7.78E-05
6	ko04921	Oxytocin signaling pathway	52(1.21%)	9.50E-05
7	ko04011	MAPK signaling pathway—yeast	18(0.42%)	0.000107
8	ko00190	Oxidative phosphorylation	61(1.42%)	0.000184
9	ko04020	Calcium signaling pathway	44(1.02%)	0.000225
10	ko03015	mRNA surveillance pathway	40(0.93%)	0.000229
11	ko04964	Proximal tubule bicarbonate reclamation	14(0.32%)	0.000263
12	ko00010	Glycolysis / Gluconeogenesis	33(0.77%)	0.000275
13	ko04721	Synaptic vesicle cycle	28(0.65%)	0.000563
14	ko04540	Gap junction	36(0.84%)	0.000615
15	ko04910	Insulin signaling pathway	47(1.10%)	0.000808
16	ko05031	Amphetamine addiction	25(0.58%)	0.000827
17	ko04152	AMPK signaling pathway	36(0.84%)	0.000843
18	ko01200	Carbon metabolism	52(1.21%)	0.000925
19	ko05152	Tuberculosis	46(1.07%)	0.001149
20	ko04151	PI3K-Akt signaling pathway	72(1.67%)	0.001306

**Fig 5:**
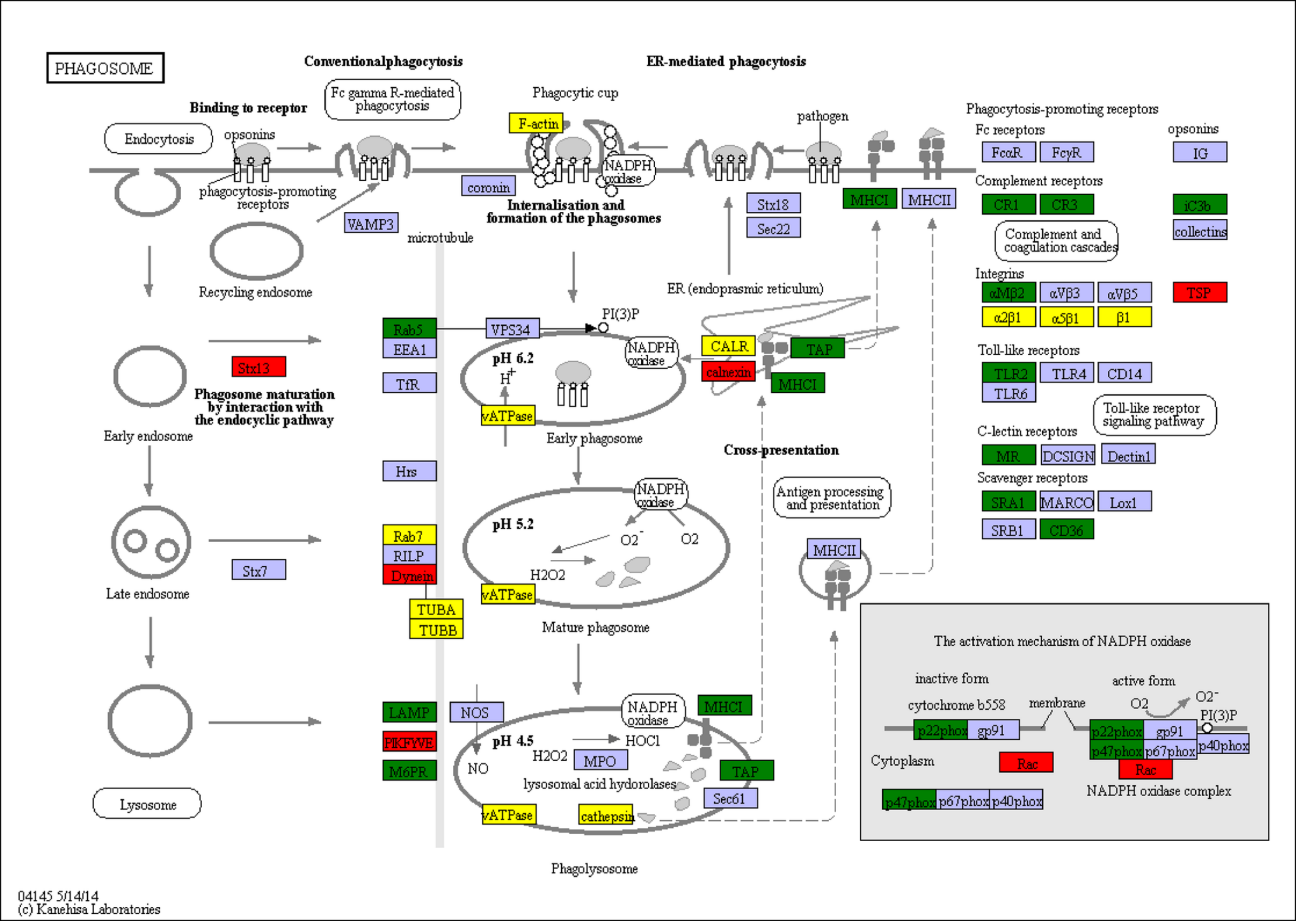
Significantly differentiated expressed genes that were identified by KEGG as involved in the PI3K/AKT signaling pathway. Red boxes indicate significantly increased expression. Green boxes indicate significantly decreased expression. Yellow boxes indicate both significantly increased and decreased expression. Blue boxes indicate unchanged expression.

In the comparison of survived samples and control, a total of 4,311 genes were found to be aberrantly expressed, with 3,308 significantly up-regulated and 1,003 significantly down-regulated (Fig 3B). In brief, among these 4,311 DEGs, 2,531 genes were expressed in both control and survived samples, including 1,825 significantly up-regulated genes and 589 significantly down-regulated genes. Moreover, 1,483 genes were only expressed in the survived samples. To confirm the biological function of DEGs, GO functional and KEGG pathway enrichment analysis for the DEGs were carried out. GO functional enrichment analysis results from the DEGs of comparison between survived samples and control indicated that 2,081 of the 4,311 DEGs had a GO ID and categorized into 6,302 functional groups in three categories (biological process, cellular component and molecular function). The top 10 terms of each category were showed in Fig 4B. Most of the GO terms were involved in the biological process, such as “positive regulation of alpha-beta T cell differentiation” and “intracellular sequestering of iron ion”, which were over-represented. The GO terms “chemokine receptor binding”, “5.8S rRNA binding” and “nucleoside kinase activity” were the three most significantly represented terms in the molecular_function category. Of the cellular component, the GO term “collagen type IV trimer”, “cell division site” and “cytosolic large ribosomal subunit” were the three most over-represented. To further investigate the biochemical pathways of DEGs, all of the DEGs from comparison between survived samples and control were mapped in the KEGG database and compared with the whole transcriptome background to search for genes refer to the innate immune response or signaling pathway. Of the 4,311 DEGs, 1,309 genes had a KO ID and were associated with 348 pathways (978 up-regulated genes categories into 334 pathways, and 331 down-regulated genes categories into 281 pathways). KEGG pathway enrichment analysis showed that 82 pathways were obviously changed (P-value < 0.05) in the survived sample compared with the control sample and the top 20 enriched pathways were showed in Table 5, with genes involved in immune-related “Phagosome”, “cGMP—PKG signaling pathway”, “MAPK signaling pathway—yeast” and “Oxidative phosphorylation” being the most significantly enriched. And 78 of the 4,311 DEGs were enriched in “Phagosome”, with 43 DEGs up-regulated and 35 DEGs down-regulated (Fig 5). Additional, some well-known immune related pathway or molecules, “mRNA surveillance pathway”, “Insulin signaling pathway”, “PI3K-Akt signaling pathway”, “NOD-like receptor signaling pathway”, “inhibitor of apoptosis proteins”, “Interleukin”, “HSPs”, “Tumor necrosis factor” and so on, were also identified through KEGG enrichment. All those DEGs were showed in S1 Table.

**Fig 6:**
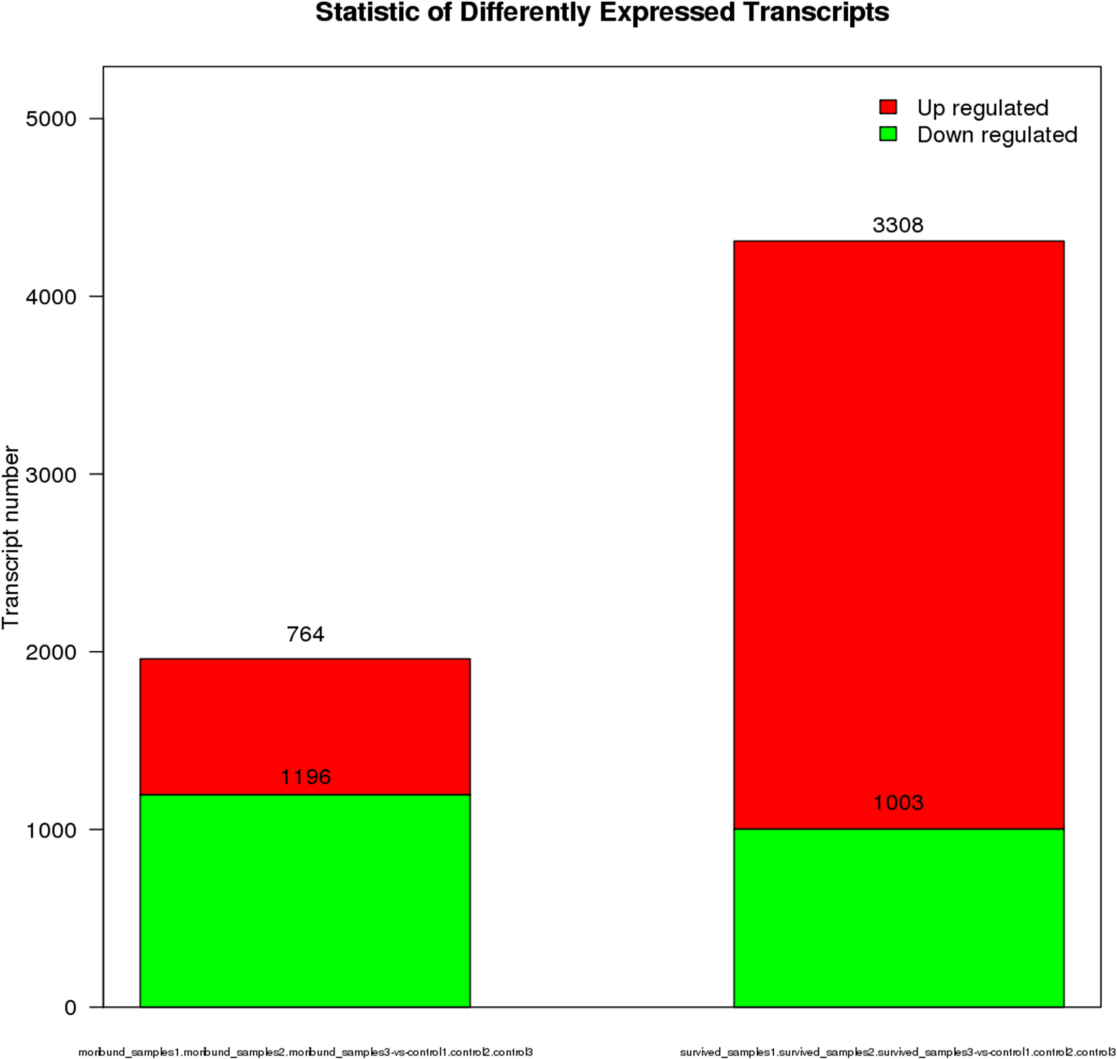
Statistic of differently expressed genes in the WSSV-infected samples with control. The first column in the chart means differently expressed gene numbers in the comparison of moribund samples and control. The second column in the chart means differently expressed gene numbers in the comparison of survived samples and control. Red parts indicate up-regulated genes and green parts indicate down-regulated genes.

**Fig 7:**
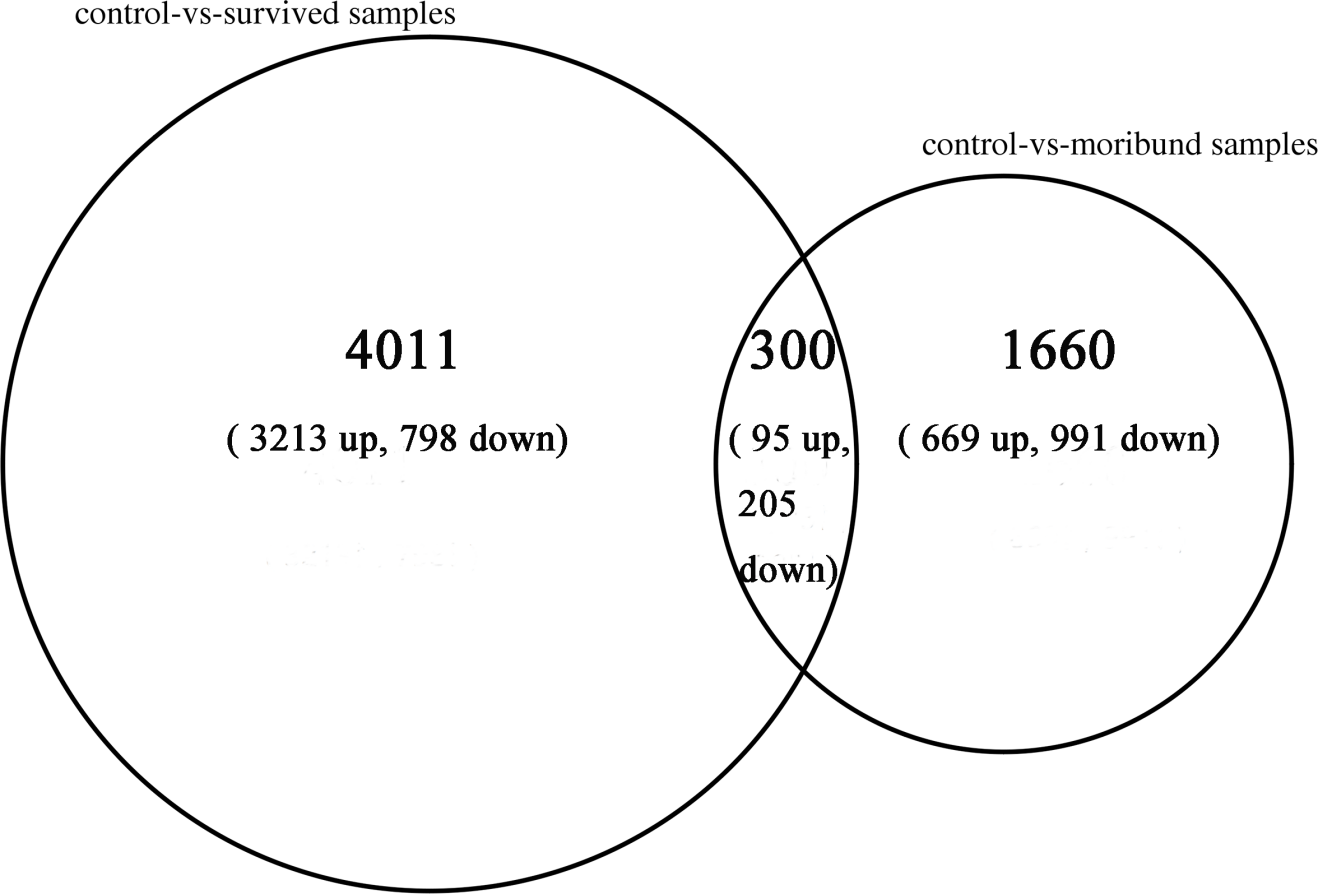
Differently expressed genes in the WSSV-infected samples with control. The left big circle represented DEGs in the comparison of survived samples and control. The right small circle represented DEGs in the comparison of moribund samples and control. Gene number was shown with up arrow and down arrow representing up-regulation and down-regulation of these DEGs, respectively. Mutual genes in the two comparison libraries were closed in the area cross two circles.

Furthermore, the number of DEGs in the comparison of survived samples and control was more than twice times than the comparison of moribund samples and control (Fig 6). In the comparison of survived samples and control, the number of up-regulated genes was more than the comparison of moribund samples and control, while the number of down-regulated genes was less than. In the contrast of two comparison libraries, 4,011 DEGs were only expressed in the comparison of survived and control library with 3,213 up-regulated genes and 798 down-regulated genes, and 1,660 DEGs were only expressed in the comparison of moribund and control library with 699 up-regulated genes and 991 down-regulated genes. The two comparison libraries were have 300 mutual DEGs with 95 up-regulated genes and 205 down-regulated genes (Fig 7).

### Validation of RNA-seq results by qRT-PCR

**Fig 8:**
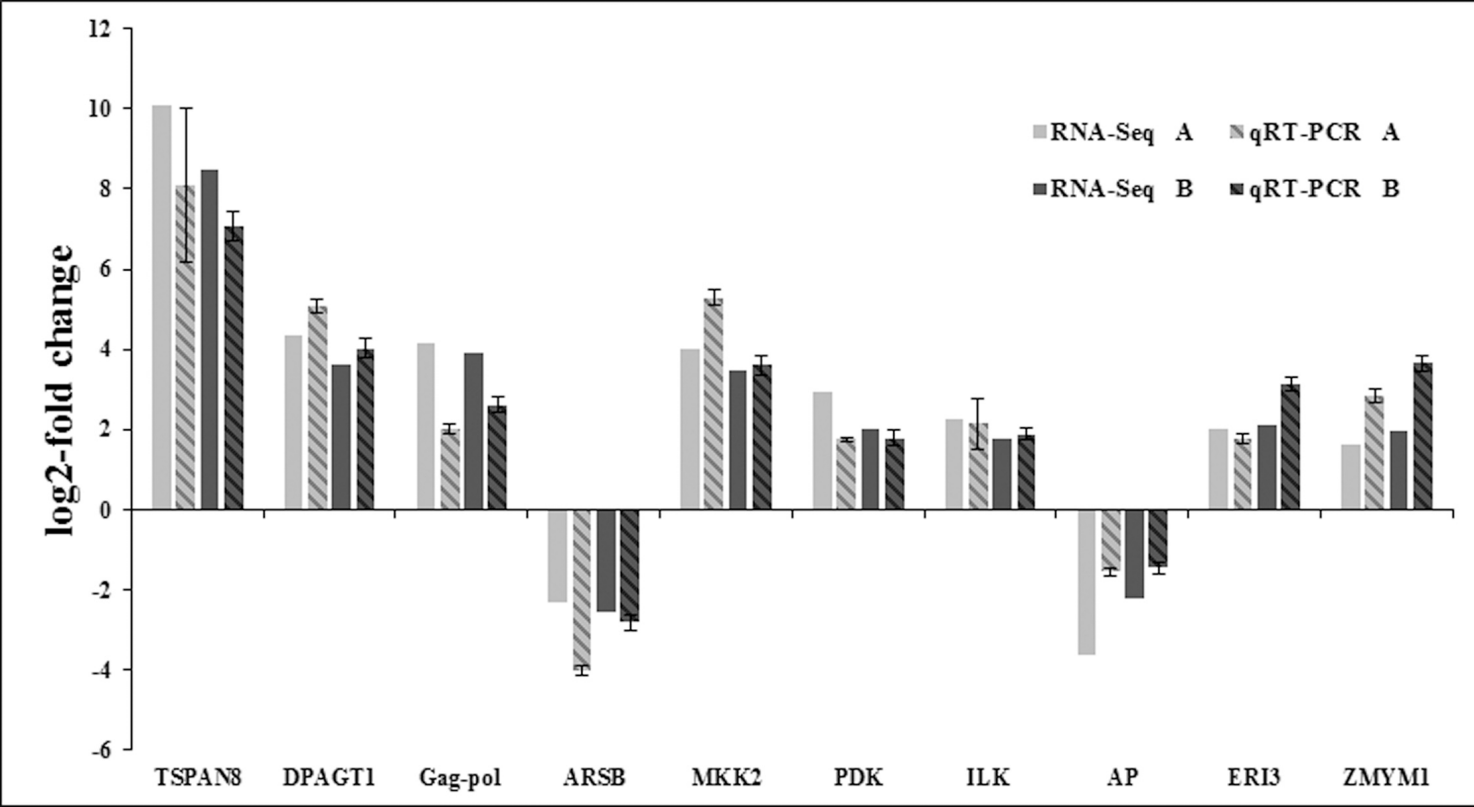
Comparison of the expression profiles of selected genes as determined by RNA-Seq (Illumina Hiseq2500^TM^ sequencing) and qRT-PCR. The letter “A” and “B” in the RNA-Seq and qRT-PCR mean the comparison of expression profiles of selected genes between control and moribund samples (the correlation coefficient was 0.92), the comparison of expression profiles of selected genes between control and survived samples (the correlation coefficient was 0.95), respectively. Target gene abbreviations are as follows: TSPAN8 –TSPAN8, DPAGT1 –UDP-N-acetylglucosamine—dolichyl-phosphate N-acetylglucosaminephosphotransferase, Gag-pol –Gag-Pol polyprotein, ARSB–arylsulfatase B, MKK2 –MAP kinase-activated protein kinase 2, PDK–pyruvate dehydrogenase (acetyl-transferring) kinase, ILK–integrin-linked protein kinase, AP–acid phosphatase, ERI3 –ERI1 exoribonuclease 3, ZMYM1 –zinc finger MYM-type protein 1. Error bars indicated standard deviations of averages from three replicates.

To further validate the results from the RNA sequencing data, ten genes were selected for qRT-PCR analysis. Specific primers were designed by the Primer Premier 3 software [52]. And the amplified fragments were sequenced for target verification. The qRT-PCR analysis of each sample were performed in three repeated. Of the ten selected genes, all showed similar expression patterns in the qRT-PCR analysis as observed from RNA-seq data (Fig 8). For example, in the RNA sequencing data of comparison between control and moribund samples validation, TSPAN8, UDP-N-acetylglucosamine—dolichyl-phosphate N-acetylglucosaminephosphotransferase, Gag-Pol polyprotein, MAP kinase-activated protein kinase 2, pyruvate dehydrogenase (acetyl-transferring) kinase, integrin-linked protein kinase, ERI1 exoribonuclease 3 and zinc finger MYM-type protein 1 were up-regulated 10.08, 4.34, 4.17, 4.01, 2.93, 2.28, 2.02 and 1.65 log_2_-fold changes respectively; and 8.09, 5.08, 2.03, 5.29, 1.75, 2.15, 1.79 and 2.85 log_2_-fold changes respectively in the qRT-PCR analysis. The arylsulfatase B and acid phosphatase genes were down-regulated by -2.29 and -3.63 log_2_-fold changes in our transcriptome data, and were similarly found to be down-regulated by -4.01 and -1.55 log_2_-fold changes in the qRT-PCRanalysis. In the RNA sequencing data of comparison between control and survived samples validation, TSPAN8, UDP-N-acetylglucosamine—dolichyl-phosphate N-acetylglucosaminephosphotransferase, Gag-Pol polyprotein, MAP kinase-activated protein kinase 2, pyruvate dehydrogenase (acetyl-transferring) kinase, integrin-linked protein kinase, ERI1 exoribonuclease 3 and zinc finger MYM-type protein 1 were up-regulated 8.48, 3.61, 3.94, 3.46, 2.03, 1.75, 2.12 and 1.99 log_2_-fold changes respectively; and 7.09, 4.03, 2.62, 3.61, 1.79, 1.89, 3.15 and 3.65 log_2_-fold changes respectively in the qRT-PCR analysis. The arylsulfatase B and acid phosphatase genes were down-regulated by -2.55 and -2.21 log_2_-fold changes in our transcriptome data, and were similarly found to be down-regulated by -2.81 and -1.45 log_2_-fold changes in the qRT-PCR analysis. The correlation coefficient of transcriptome data and qRT-PCR in two comparison libraries was 0.92 and 0.95, respectively. Although the results from both analyses didn’t match perfectly, perhaps due to sequencing biases [40], the qRT–PCR analysis substantially confirmed the direction of changes obtained from the RNA sequencing analysis.

### The responses of immune-related genes in *M*. *nipponense* to WSSV challenge

Numerous immune-related genes were obtained from two comparison libraries, the library of comparison between control and moribund samples and the library of comparison between control and survived samples. In total, 88 immune-related genes were obtained. These immunes genes are groups into 13 functions (Table 6), Which include antimicrobial (1 genes), Proteases (11 genes), protease inhibitors (2 genes), cell death (5 genes), cell adhesion (9 genes), heat shock proteins (2 genes), oxidative stress (10 genes), pathogen recognition immune receptors (4 genes), signaling pathway(Wnt signaling pathway-5 genes, PI3K/AKT/mTOR pathway-3 genes, MAPK signaling pathway-17 genes, JAK/STAT pathway-3 genes, Ubiquitin Proteasome Pathway-6 genes) and other immune genes (20 genes).

**Table 6:** Candidate genes involved in *M*. *nipponensis* immune response.

Candidate genes involved in the comparison of survived samples with control			
Category or gene ID	Homologous function	Species	FC^a^
*Proteases*			
CL11768Contig1	cathepsin S	*Mus musculus*	-3.17
CL7706Contig1	cathepsin B1	Clonorchis sinensis	8.68
comp79933_c0_seq1_2	cathepsin B	*Schistosoma japonicum*	7.91
CL1Contig9793	Cathepsin C	*Schistosoma haematobium*	8.15
CL18412Contig1	Cathepsin L precursor	*Schistosoma japonicum*	7.34
comp35611_c0_seq3_2	26S protease regulatory subunit, putative	*Schistosoma mansoni*	7.41
*Proteases inhibitor*			
CL6002Contig1	serine protease inhibitor A3G	*Mus musculus*	-5.97
comp75245_c0_seq2_2	serine protease inhibitor	*Paragonimus westermani*	Inf
Signal transduction			
*Wnt signalling pathway*			
comp32201_c0_seq1_2	ring box protein	*Schistosoma mansoni*	6.98
comp86621_c0_seq3_2	casein kinase 1 epsilon	*Eurydice pulchra*	2.54
CL16567Contig1	casein kinase I isoform delta	*Clonorchis sinensis*	9.01
CL13787Contig1	Casein kinase II subunit beta	*Opisthorchis viverrini*	6.05
comp77179_c0_seq6_2	casein kinase I isoform gamma-3	*Clonorchis sinensis*	7.37
*PI3K-Akt-mTOR pathway*			
comp16015_c0_seq1_2	regulatory associated protein of mTOR	*Opisthorchis viverrini*	Inf
comp36351_c0_seq1_2	FKBP12-rapamycin complex-associated protein	*Clonorchis sinensis*	Inf
comp36351_c1_seq1_2	ataxia telangiectasia mutated (atm)-related	*Schistosoma mansoni*	Inf
*MAPK signalling pathway*			
CL10155Contig1	Activating transcription factor 4	*Mus musculus*	-3.16
comp87614_c0_seq6_2	serine/threonine-protein kinase PLK4-like	Hyalella azteca	2.37
CL4900Contig1	serine/threonine-protein kinase Sgk1 isoform c	Mus musculus	-3.67
comp49509_c0_seq1_2	Serine/threonine-protein kinase mig-15	*Schistosoma haematobium*	Inf
comp78464_c0_seq1_2	serine/threonine-protein kinase Nek4 isoform X1	*Chinchilla lanigera*	Inf
comp79342_c0_seq3_2	serine/threonine-protein kinase PRP4	*Clonorchis sinensis*	9.14
comp71890_c0_seq3_2	serine/threonine-protein kinase 25	*Clonorchis sinensis*	8.91
CL17850Contig1	serine/threonine-protein kinase par-1	*Clonorchis sinensis*	8.09
comp78560_c0_seq3_2	serine/threonine-protein kinase NIM1	*Clonorchis sinensis*	7.48
comp65802_c0_seq1_2	serine/threonine-protein kinase TTK/MPS1	*Clonorchis sinensis*	7.36
CL2265Contig2	c-Jun N-terminal kinase	*Clonorchis sinensis*	7.10
comp72868_c0_seq2_2	MAP kinase phosphatase	*Schistosoma mansoni*	7.00
CL1Contig13196	MAP kinase-activated protein kinase 2	*Eriocheir sinensis*	3.46
comp77070_c0_seq6_2	extracellular signal-regulated kinase 1/2	*Scylla paramamosain*	9.04
CL14855Contig1	Mitogen-activated protein kinase (MAPK) kinase MKK4	*Schistosoma mansoni*	7.69
comp81318_c0_seq5_2	Max protein	*Opisthorchis viverrini*	6.58
*JAK/STAT pathway*			
comp76201_c0_seq1_2	growth factor receptor-binding protein 2	*Clonorchis sinensis*	7.25
*Cell death*			
CL1Contig6129	CASP8 and FADD-like apoptosis regulator	*Mus musculus*	-3.79
comp78381_c0_seq11_2	run domain Beclin-1 interacting and cystein-rich containing protein	*Clonorchis sinensis*	7.07
CL20513Contig1	programmed cell death	*Schistosoma mansoni*	8.31
comp88816_c0_seq1_2	prohibitin	*Schistosoma mansoni*	9.96
*Cell adhesion*			
comp34968_c0_seq1_2	ADP-ribosylation factor-like 6	*Clonorchis sinensis*	8.94
comp83430_c3_seq9_2	ADP-ribosylation factor-binding protein GGA1	*Schistosoma japonicum*	9.32
CL7706Contig1	ADP-ribosylation factor	*Schistosoma mansoni*	8.68
comp34891_c0_seq2_2	ADP-ribosylation factor-like 3	*Schistosoma japonicum*	7.86
CL18876Contig1	adp-ribosylation factor 2	*Ascaris suum*	7.60
comp35052_c0_seq1_2	ADP-ribosylation factor-like protein 2-binding protein	*Clonorchis sinensis*	7.55
comp79928_c0_seq3_2	integrin	*Schistosoma mansoni*	9.57
*Heat shock proteins*			
comp87970_c0_seq2_2	heat shock protein 21	*Macrobrachium rosenbergii]*	3.50
CL11343Contig1	heat shock protein 70	*Penaeus monodon*	-3.15
*Oxidative stress*			
CL12763Contig1	thioredoxin	*Mus musculus*	-2.96
comp32414_c0_seq1_2	peroxiredoxin 3	*Clonorchis sinensis*	9.88
CL10956Contig1	peroxiredoxin-1	*Mesocricetus auratus*	-3.23
CL18982Contig1	microsomal glutathione S-transferase 1 isoform 2	*Mus musculus*	-3.09
comp60048_c0_seq1_2	Mn-SOD	*Paragonimus westermani*	8.75
comp32905_c0_seq1_2	cytosolic Cu/Zn superoxide dismutase	*Paragonimus westermani*	7.24
*PPRs*			
CL10185Contig1	C-type lectin domain family 4, member e	*Mus musculus*	3.63
CL10259Contig1	C-type lectin domain family 4, member d	*Mus musculus*	3.62
*Other immune genes*			
CL20347Contig1	metallothionein 2	*Mus musculus*	-4.32
CL10847Contig1	profilin 1, isoform CRA_c	*Rattus norvegicus*	-4.31
CL17138Contig1	Selenoprotein W	*Schistosoma mansoni*	7.62
CL681Contig3	selenoprotein-like	*Hyalella azteca*	-3.74
CL10615Contig1	Selenoprotein X1	*Mus musculus*	-3.97
comp88625_c0_seq1_2	soma ferritin-like	*Limulus polyphemus*	10.02
comp66351_c0_seq1	chitinase 4	*Macrobrachium nipponense*	-2.90
CL1Contig9190	chitinase 3C	*Macrobrachium nipponense*	-3.60
comp74350_c0_seq6_2	Actin-related protein 6	*Schistosoma japonicum*	8.73
CL21493Contig1	actin	*Schistosoma japonicum*	7.55
CL10277Contig1	calponin-3	*Clonorchis sinensis*	7.67
comp69528_c0_seq3_2	Calmodulin, partial	*Schistosoma haematobium*	7.54
comp61515_c0_seq1	interferon regulatory factor	*Litopenaeus vannamei*	Inf
CL10224Contig1	complement factor B	*Felis catus*	-3.19
CL1201Contig1	complement C3 isoform X1	*Bos taurus*	-3.21
CL12167Contig1	tumor necrosis factor ligand 1	*Mus musculus*	-3.22
**Candidate genes involved in the comparison of moribund samples with control**			
**Category or gene ID**	**Homologous function**	**Species**	**FC**^a^
*Antimicrobial*			
CL1794Contig1	crustin	*Macrobrachium rosenbergii*	-3.80
*Proteases*			
CL1Contig6818	cathepsin C	*Fenneropenaeus chinensis*	-2.76
CL3395Contig1	cathepsin D	*Palaemon carinicauda*	-2.32
CL10339Contig1	cathepsin L	*Pandalus borealis*	-2.15
CL11978Contig1	cathepsin A	*Eriocheir sinensis*	5.92
*MAPK signalling pathway*			
CL1Contig13196	MAP kinase-activated protein kinase 2	*Scylla paramamosain*	4.01
*Cell death*			
CL440Contig1	inhibitor of apoptosis protein	*Palaemon carinicauda*	1.73
*Cell adhesion*			
comp64523_c0_seq2	ADP-ribosylation factor-like protein 6	*Orchesella cincta*	9.47
CL1514Contig2	integrin alpha 4	*Fenneropenaeus chinensis*	-4.09
*Oxidative stress*			
comp85041_c1_seq7_2	thioredoxin-like protein 4A isoform X2	*Diaphorina citri*	3.03
CL22077Contig1	glutathione S-transferases	*Macrobrachium nipponense*	-1.66
CL13799Contig1	glutathione peroxidase-like	*Priapulus caudatus*	-1.46
CL153Contig3	copper/zinc superoxide dismutase isoform 4	*Marsupenaeus japonicus*	4.12
*Heat shock proteins*			
comp64778_c0_seq3	heat shock protein 21	*Macrobrachium rosenbergii*	3.46
CL3679Contig1	heat shock protein 70	*Trichoplusia ni*	-4.40
*PPRs*			
comp67093_c1_seq1_3	C-type lectin 2	*Fenneropenaeus chinensis*	-2.46
CL14655Contig1	lipopolysaccharide and beta-1,3-glucan binding protein	*Macrobrachium nipponense*	19.33
*Other immune genes*			
CL1Contig9190	chitinase 3C	*Macrobrachium nipponense*	-3.52
comp40522_c0_seq1_3	chitinase 1	*Macrobrachium nipponense*	-2.61
comp64036_c1_seq4_3	chitinase 3B	*Macrobrachium nipponense*	-2.31
CL1Contig3188	adenosine deaminase CECR1-like	*Branchiostoma belcheri*	-1.89

Inf means the gene only expressed in the survived samples.

^a^ Fold changes (log_2_ ratio) in gene expression.

PRPs-pattern recognition proteins, *Pro*PO-prophenoloxidase.

**Fig 9:**
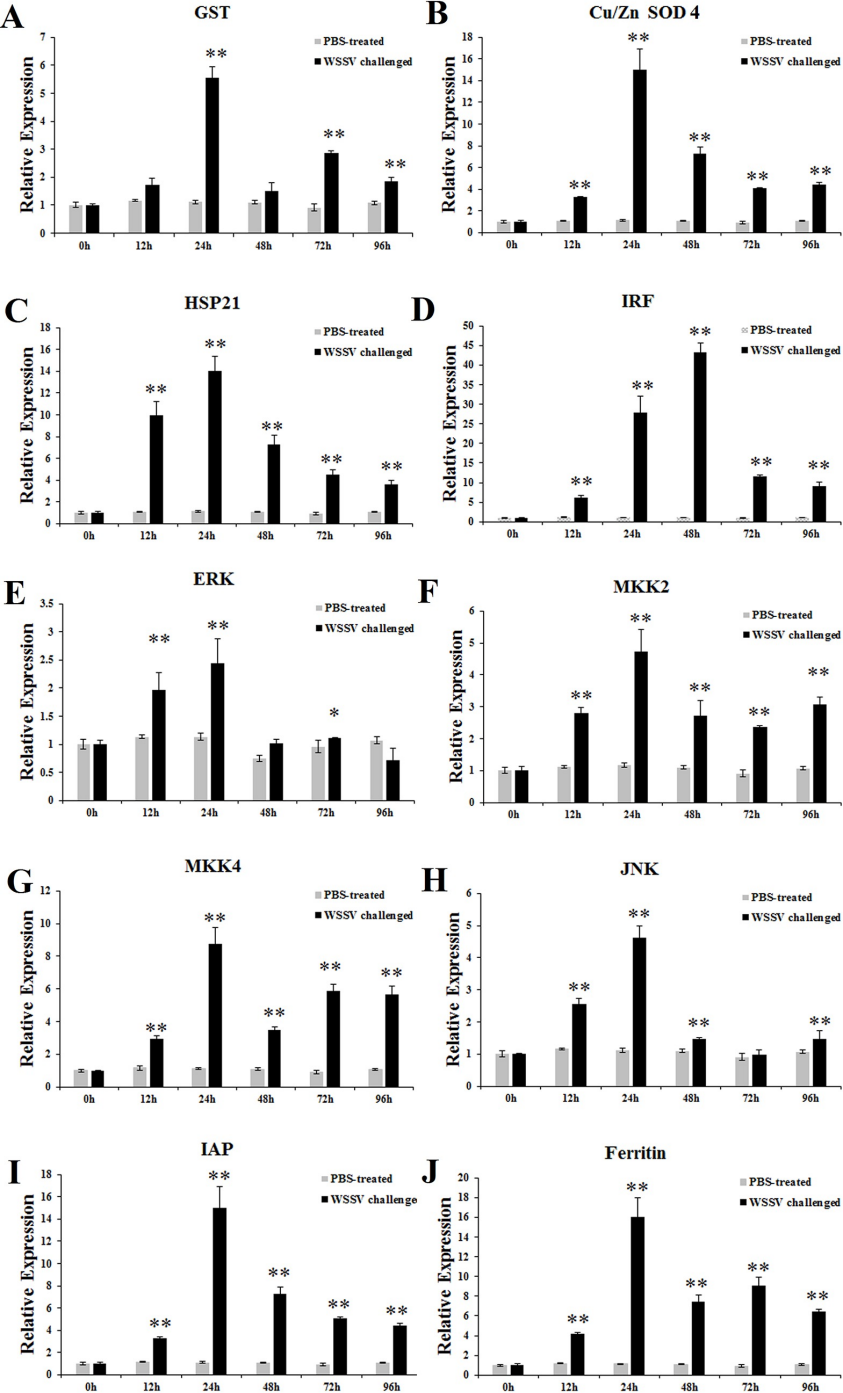
Expression profiles of genes in hepatopancreas from WSSV challenged *M*. *nipponensis*. Expression profiles of GST (A), Cu/ZnSOD 4 (B), HSP21 (C), IRF (D), ERK (E), MKK2 (F), MKK4 (G), JNK (H), IAP (I) and Ferritin (J) in hepatopancreas from WSSV challenged *M*. *nipponensis*. Real-time RT-PCR was performed in triplicate for each sample. Expression values were normalized to those of β-actin using the Livak (2^-ΔΔCT^) method and the data were provided as the means ± SD of triplicate assays. Expression level detected at 0 h was set as 1.0. Statistical significance was determined by Student's t-test (* indicates P < 0.05, ** indicates P < 0.01).

The time-course mRNA expression changes of ten genes in hepatopancreas tissue after WSSV challenge were investigated by qRT-PCR. The ten genes were glutathione s-transferase (GST), copper/zinc superoxide dismutase 4 (Cu/Zn SOD 4), heat shock protein 21 (HSP21), interferon regulatory factor (IRF), extracellular signal-regulated kinases 1/2 (ERK), MAP kinase-activated protein kinase 2 (MKK2), MAP kinase-activated protein kinase 4 (MKK4), c-Jun N-terminal kinase (JNK), inhibitor of apoptosis protein (IAP) and ferritin. As shown in Fig 8. Upon WSSV challenge, the transcriptional level of GST was significantly up-regulated at 12 h (~1.74-fold), and maintained a high level in the whole stage of treatment with a most remarkable value (~5.54-fold) at 24 h, and then drop to basic level at 48 h (Fig 9A). During WSSV challenge, the expression of Cu/Zn SOD 4 was dramatically up-regulated at 12 h with ~3.26-fold, and then a high level with a peak value of ~15.04-fold at 24 h (Fig 9B). In reponse to WSSV challenge, the transcript level of HSP21 was up-regulated during 12–96 h with ~9.99-fold, ~14.00-fold, ~7.28-fold, ~4.54-fold and ~3.61-fold, respectively (Fig 9C). After WSSV treatment, the IRF expression was significantly enhanced at 12–96 h with the first peak of ~6.27-fold at 12 h and the second peak of ~43.16-fold at 48 h, respectively (Fig 9D). With the infection of WSSV, the expression of ERK showed a raise at 12 h and 24 h with ~1.97-fold and ~2.43-fold, respectively (Fig 9E). In WSSV treated group, MKK2 mRNA was aroused at 12, 24, 48, 72 and 96 h, with~2.79-fold, ~4.73-fold, ~2.72-fold, ~2.37-fold and ~3.08-fold, respectively (Fig 9F). With exposure to WSSV, MKK4 expression increased at 12–96 h with the first peak at 12 h with ~2.95-fold, the second peak at 24 h with ~8.78-fold, and the third peak at 72 h with ~5.85-fold, respectively (Fig 9G). After WSSV infection, the expression of JNK rapidly increased at 12 h with ~2.55-fold and 24 h with ~4.61-fold, and then dropped to ~1.46-fold at 48 h, and near the control level at 72 h, and finally increased again at 96 h with ~1.47-fold, respectively (Fig 9H). During WSSV infection, the transcript level of IAP was up-regulated from 12 h with ~3.26-fold, dramatically increased at 24h with ~15.04-fold, and then dropped to ~7.26-fold, ~5.10-fold, and ~4.46-fold at 48, 72, and 96h, respectively (Fig 9I). For WSSV challenge, the transcript level of ferritin was significantly up-regulated at 12 h (~4.16-fold), and maintained a high level in the whole stage of treatment with a most remarkable value (~16.05-fold) at 24 h (Fig 8J). Regarding the mRNA levels of ten genes in hepatopancreas, there is no significantly change from 12 h to 96 h during PBS injection.

## Discussion

White spot syndrome virus (WSSV) is one of the most serious pathogens of the cultured commercial shrimps, responsible for high mortality and consequent economic losses to shrimp industry worldwide [16, 32]. Knowledge about the interaction between *M*. *nipponense* and the virus is limited, in spite of plenty published studies on the characterization and detection of WSSV available [16, 53–56]. A better understanding of viral-host interaction is imminently needed to help develop a pro-active approach to fighting and suppressing this virus.

As a fast and cost-efficient research tool for de novo assembled analysis of transcriptome, next-generation sequencing technology has been rapidly developed and utilized to discover novel genes as well as to identify the expression levels of genes in non-model organisms which lack a complete genome database successfully [57, 58]. Some transcriptome studies on *M*. *nipponense* have been performed [59–61]. This study was conducted to identify some immune-related genes and pathways that was associated with WSSV resistance in the hepatopancreas of *M*. *nipponense*. In the present study, hepatopancreas of the healthy, moribund and survived prawns were separately collected and used to performed the high-throught sequencing.

A total of 64,049 predicted unigenes with N50 length of 2,205 bp were obtained. Based on the annotation information, 16,573 unigenes (25.88% of total unigenes) were classified into 63 functional groups, where 49.10% comprised biological processes, followed by cellular component(38.98%) and molecular function (11.92%). Genes involved in “cell process” (20.22%), “single-organism process” (16.99%), and “metabolic process” (16.26%) accounted for a large proporition of biological processes. The species distribution analysis showed that these unigenes shared 19.81% similarity to *H*. *azteca*, and 50.56% unigenes did not have high homology with other species. This result may have been caused by a lack of genomic information. KEGG pathway analysis was applied to predict gene function. A total of 7,759 unigenes were mapped to obtain a total of 376 biological pathways, including some infectious diseases and immune signaling pathways. These predicted biological processes and pathways can provide useful information for deeper investigation of gene functions. Better understanding of the innate immune abilities and immune defense mechanisms of shrimp will be beneficial to the development of health management and disease control in prawn aquaculture [62].

For crustaceans like *M*. *nipponense*, the innate immune system is central to defend themselves against various pathogens and environmental stress and the first step of innate immunity is pattern recognition. This defence system is launched by checking the invading pathogen through pattern recognition receptors (PRPs), which can recognize pathogen-associated molecular patterns (PAMPs) and activate the host immune response [63]. To date, 11 kinds of pattern recognition receptors (PRRs) have been identified in shrimp, containing β-1,3-glucan-binding protein, β-1,3-glucanase-related protein, C-type lectin, galectin, scavenger receptor, fibrinogen-related protein, thioester-containing protein, Down syndrome cell adhesion molecule, serine protease homolog, toll-like receptor, and *rans*-activation response RNA-binding protein [64]. Four genes in three PPRs (C-type lectins, galectin and scavenger receptor) expressions were affected by WSSV treatment in our transcriptome data.

**Fig 10:**
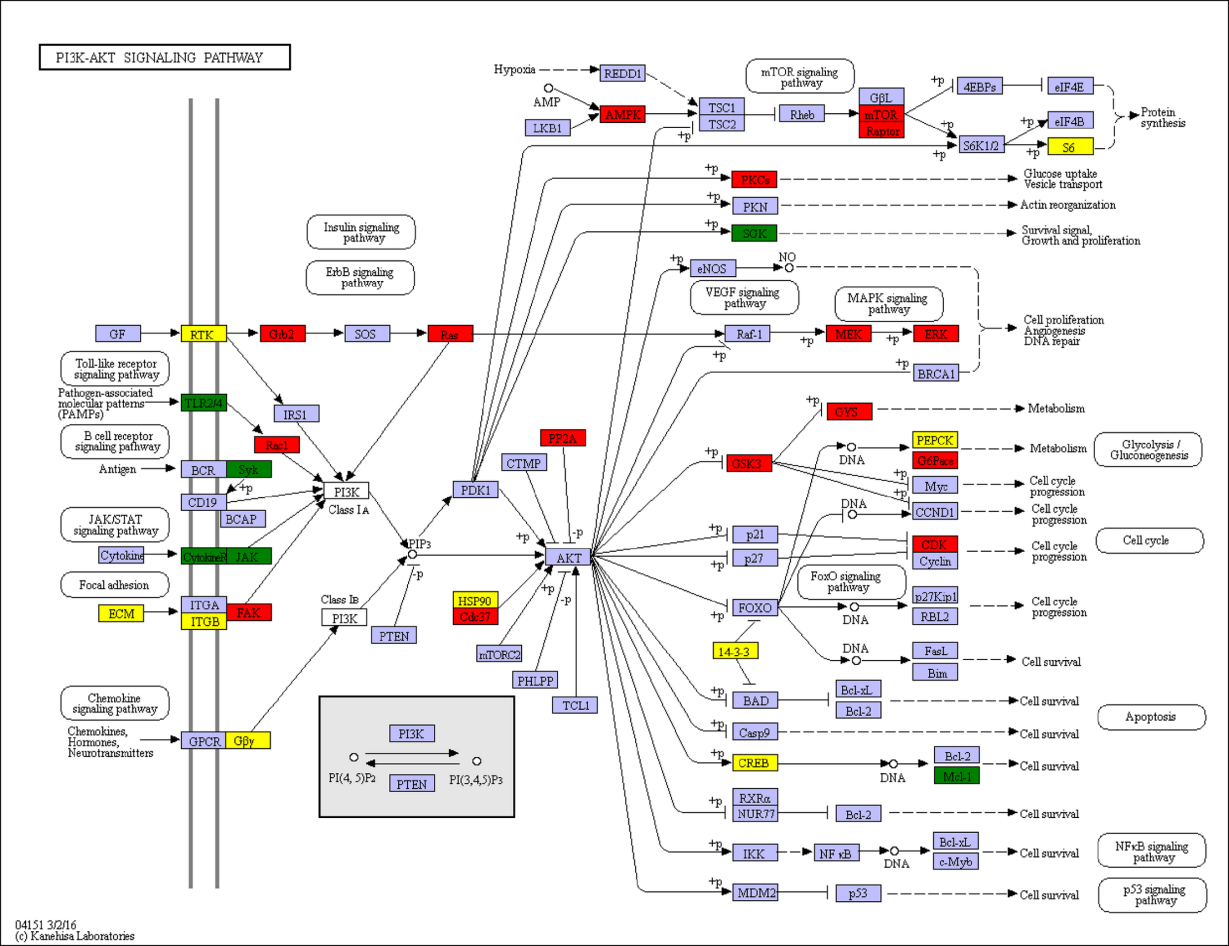
Significantly differentiated expressed genes that were identified by KEGG as involved in the PI3K/AKT signaling pathway. Red boxes indicate significantly increased expression. Green boxes indicate significantly decreased expression. Yellow boxes indicate both significantly increased and decreased expression. Blue boxes indicate unchanged expression.

The PI3K/AKT/mTOR pathway is known to be one of the three major signaling pathways in regulating the cell cycle. The PI3K-Akt signaling pathway can be activated by many types of cellular stimuli or toxic insults and regulates fundamental cellular functions such as transcription, translation, proliferation, growth, and survival. Activation of the PI3K/AKT signaling pathway is common to viral infection, and many virus manipulated this signaling pathway to ensure successful virus replication [65]. mTOR is a key kinase downstream of PI3K/AKT [66]. PI3K/AKT/mTOR pathway was activated by WSSV in *L*. *vannamei* and *Procambarus clarkii* [65–67]. In our transcriptome data, PI3K/AKT/mTOR pathway was also activated, three unigenes identified as important components PI3K/AKT/mTOR pathway, regulatory associated protein of mTOR (Raptor), FKBP12-rapamycin complex-associated protein and ataxia telangiectasia mutated (atm)-related were highly expressed (Fig 10).

**Fig 11:**
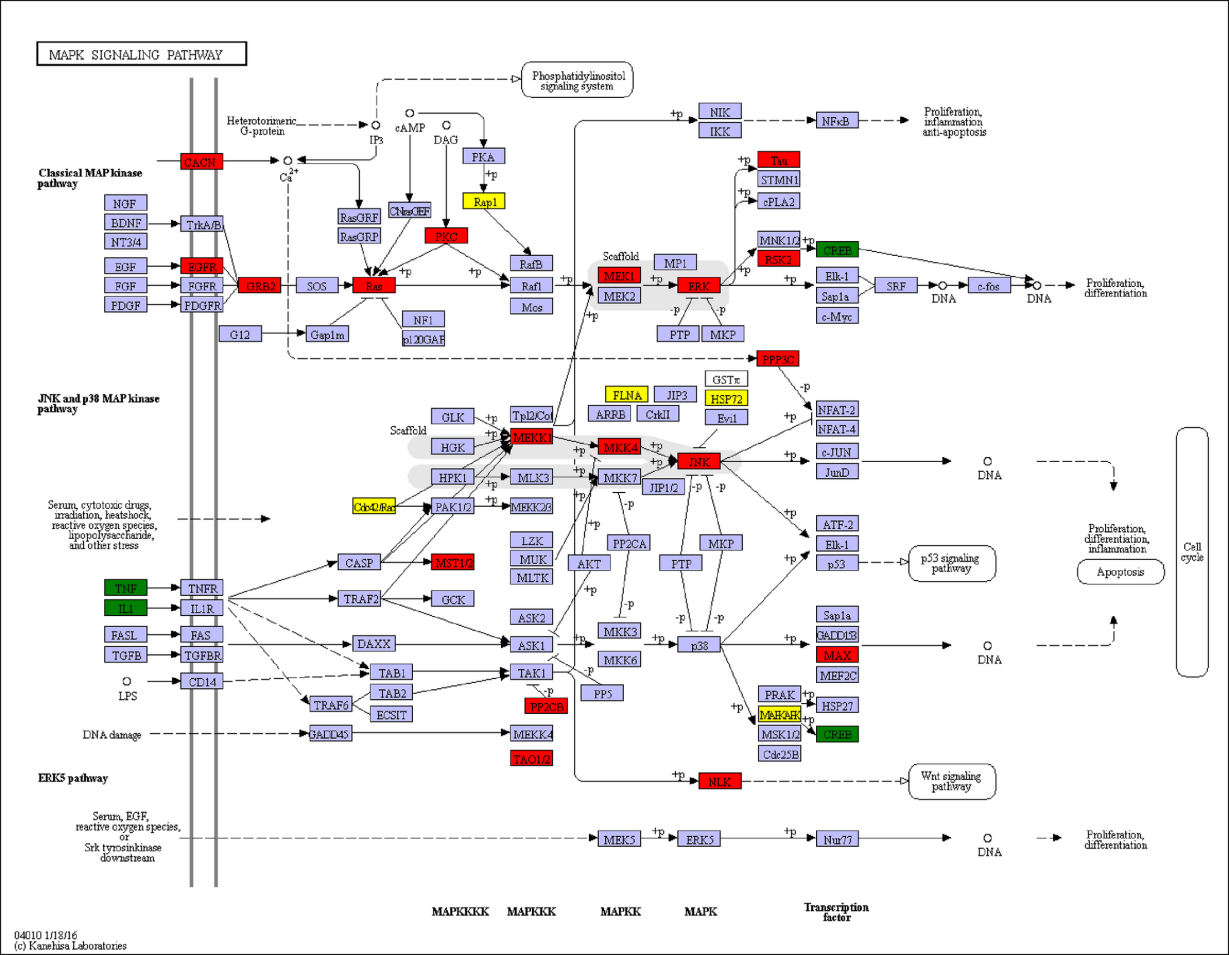
Significantly differentiated expressed genes that were identified by KEGG as involved in the MAPK signaling pathway. Red boxes indicate significantly increased expression. Green boxes indicate significantly decreased expression. Yellow boxes indicate both significantly increased and decreased expression. Blue boxes indicate unchanged expression.

MAPK signaling pathway play a very important role in cellular response to extracellular stimuli, including inflammatory cytokines, environmental stress and pathogenic infection [68]. In vertebrates, MAPK modules are involved in both innate and adaptive immunity processes [69, 70]. In invertebrates, MAPK modules have been demonstrated to be one of crucial signal pathways regulating innate immune system, especially in the immune process against pathogenic infection [71]. MAPK was divided into three major groups: extracellular signal-regulated kinases (ERK), c-Jun N-terminal kinase (JNK), and p38 MAPK [72]. Each MAPK is activated by dual phosphorylation on threonine and tyrosine with a Thr-Xaa-Tyr motif. Generally, one MAPKK activates one specific MAPK. In *L*. *vannamei*, ERK was found to be induced activation by WSSV in the early period [73], and MKK4 was found to act an anti-virus role and is capable of phosphorylating p38 in shrimp [74]. With WSSV challenge, the mRNA expression of MKK7 was acutely up-regulated and MKK7, like counterparts in *Drosophila* and mammals, was an activator of JNK [75]. It has been demonstrated that JNK activation was involved in WSSV infection, and JNK expression increased after WSSV treatment [76]. These results suggest that this pathway plays an important role in antiviral innate immune response. In the present study, 39 genes were significantly differentially expressed in the MAPK signaling pathway, including 31 significantly up-regulated genes and 8 significantly down-regulated genes (Fig 11). To further ascertain the role of MAPK signaling pathway in antiviral immune response, four key DEGs (ERK, MKK2, MKK4 and JNK) involved in the signaling pathway were selected for the time-course qRT-PCR analyse. During WSSV time-course infection, ERK, MKK2, MKK4 and JNK were all up-regualted and all reach a peak value at 24 h in hepatopancreas of *M*. *nipponense* (Fig 9E–9H). The results were in line with previous studies in *F*. *chinensis* and *L*. *vannamei* challenged with WSSV [75, 77].

Apoptosis also plays important roles in regulating the innate cellular response through minimizing virus replication and inhibiting the spread of offspring virus in the host [32]. Caspase is an effector molecule that mediates the apoptotic process. The IAPs modulates the apoptosis mechanism by blocking the activity of caspase [32]. Nevertheless, IAPs were also demonstrated to participate in signal transduction regulating in immune reponse in insects and mammals [78–80]. IAPs were highly expressed in our transcriptome data, in *M*. *rosenbergii* and *L*. *vannamei* challenged with WSSV [32, 81]. The mRNA expression level at different time-course in hepatopancreas of *M*. *nipponense* also showed the same trend with previous reports in *M*. *rosenbergii* and *L*. *vannamei* (Fig 9I). The results means IAPs perhaps play a protective role against WSSV infection in *M*. *nipponense*.

Heat shock protein (HSP) is a family of proteins that ubiquitously distribute and highly conserve, behaving as molecular chaperones and exerting a protective effect against various stimuli [82–84]. The stimuli include heat shock, ultraviolet light, infection, inflammation, and cellular toxins. HSPs are named according to their molecular weights. For example, HSP70, HSP90, and HSP100 are families of HSPs with sizes of 70, 90, and 100 kDa, respectively. HSP proteins have six classes, HSP33, HSP60, HSP70, HSP90, HSP100 and small heat-shock proteins (sHSPs) [85]. Current researches indicated that HSPs function as regulators in the immune response and activators in the innate and acquired immunity [86]. HSPs are essential for immune responses, apoptosis and exhibit a complex interaction with viruses. A lot of HSP genes involved in shrimp immunity have been investigated: *L*. *vannamei* HSP60, HSP70, and HSP90, *F*. *chinensis* HSP70 and HSP 90, *P*. *monodon* HSP21, HSP70, and HSP90, and *M*. *japonicus* HSP40, HSP 70, and HSP 90 [87, 88]. In *E*. *carinicauda*, HSP70 and HSP90 were increase remarkably after WSSV infection in hepatopancreas [89]. In *L*. *vannamei*, down-regulation Heat shock 70 kDa protein cognate 5 (LvHSC70-5) could increase the cumulative mortality after WSSV infection and LvHSC70-5 contribute to WSSV toleration [84]. The HSP21 and HSP70 were found in our transcriptome data and changed significantly. In the present study, mRNA express level of HSP21 showed a clear time dependent response in hepatopancreas of *M*. *nipponense* after WSSV infection, indicating HSP21 could be induced by WSSV (Fig 9C). The results were accordance with previous studies in *L*. *vannamei* [90, 91] and *M*. *rosenbergii* [92], suggesting that HSP21 was involved in the reponse to WSSV infection and may play an important role in anti-virus responses in *M*. *nipponensis*.

In addition, the activation of ubiquitin proteasome pathway (UPP) in *M*. *nipponense* was observed in our transcriptome data. The UPP pathway modulates degradation of short-lived proteins in different cellular process, including signal transduction, antigen presentation and induction of the inflammatory response and apoptosis in vertebrate [93]. This pathway also participated in viral infection and assists activities required of different aspect of virus life cycle, such as entry, replication and shedding [94]. Recently, various reports have been conducted showing the hijack of UPP pathway to regulate cellular intrinsic antiviral activities and innate immunity induced by WSSV [95, 96]. E3 ubiquitin-protein ligase HUWE1 with the highest up changes after WSSV chanllenge was found in *M*. *rosenbergii* transcriptome data [32], while RING finger protein 38 was the highest up changes (with ~10.15-fold) in our data. The highly expression of E3 ubiquitin-protein ligase HUWE1 may be hijacked by WSSV to gain its own proteins. Further study is needed to fully understand the interaction between WSSV and those proteins.

Reactive oxygen species (ROS) are essential produced by oxidative stress generated in crustaceans as a defense mechanism against microbial infection [97]. Although ROS play a vital role in a host’s defense, the mass accumulation and residuals of ROS in animals will cause severe irreversible cell damage, resulting cell death [98, 99]. To protect themselves against damages of ROS, protect cells against oxidative stress and prevent or repaire oxidative damage, most cells have protective mechanisms to balance the concentration of ROS. A set of antioxidant defense systems have been developed by aerobic organisms to prevent the deleterious effects of ROS, including antioxidant enzymes such as glutathione S-transferase (GST), superoxide dismutase (SOD), catalase (CAT) and glutathione peroxidase (GPx) [100, 101]. When pathogens enter into the body of the prawn, they will be detected as invaders and intiate the innate immune systems, decrease the activity of antioxidant enzymes and release of ROS, causing oxidative stress [102, 103]. As one of the important endogenous antioxidant protein, GST provides the primary and vital line of defense against peroxide, superoxide radicals, and hydrogen peroxide [104]. GST have been proved that it play important roles in detoxification and protection from oxidative stress injury in aquatic organisms [62]. In *Exopalaemon carinicauda*, after WSSV challenge, *Ec*GST mRNA expression in hepatopancreas were up-regulated and significant rise at different time with a peak value at 6 h [101]. In *Macrobrachium rosenbergii*, *Mr*GSTD and *Mr*GSTK were up-regulated and significant rise at different time-course by WSSV with the most remarkable value at same time (48 h) [62]. Alouthgh the expression of GST was found to be down-regulated in our transcriptome data, GST was significantly changed in three comparision libraries. This phenomenon showed GST expression could easily affect by WSSV in hepatopancreas of *M*. *nipponense*. To better understand the function of GST in the immune response to WSSV in *M*. *nipponensis*, different time-course mRNA expression of GST in hepatopancreas was performed, showing up-regulated at 12 and 24 h, and then drop to basic level at 48 h (Fig 9A). The observation in this study was in accordance with the previous report in *F*. *chinensis*, *E*. *carinicauda*, *M*. *rosenbergii* [62, 101, 105]. The results of GST expression in WSSV challenge implied that it might be involved in antiviral responses in *M*. *nipponensis*.

SODs are used as biomarkers and the activities of them are intimate correlation with the immune stimulation, disease, and healthy status in aquatic organisms [106–108]. As a kind of metalloenzymes in animals, SODs were categorized into two groups according to the metal co-factor they harbor: copper-zinc SODs (Cu/Zn SOD) and manganese SODs (MnSOD) [103].The first step of ROS eliminated from cells is conducted by SODs. They can convert the superoxide radical to molecular oxygen and hydrogen peroxide that passes freely through membranes [109]. The increased SOD activity is a part of the adaptive immunity to oxidative stress [110]. In *M*. *nipponense*, with exposion to Microcystin-LR, the mRNA expression of Cu/Zn SOD was up-regulated with a peak value at 24 h and the Cu/Zn SOD activity was increased with the most remarkable value at 48 h [18]. In *L*. *vannamei*, after *Vibrio alginolyticus* injection, the mRNA expression level of ecCu/ZnSOD ascended at 3 h in hepatopancreas, demonstrated that the increase of the ROS induced by the *V*. *alginolyticus* injection lead to the up-regulation of ecCu/ZnSOD [103]. The cytosolic Cu/ZnSOD, MnSOD and Cu/ZnSOD 4 was up-regualted after WSSV infection in our transcriptome data. The increased expression of SODs in our transcriptome data might imply that they have an antiviral role against WSSV. The Cu/ZnSOD 4 was notable in the all comparision libraries. During WSSV challenge from 0 to 96 h post-injection, mRNA expression level of Cu/ZnSOD 4 was significantly up-regualted with a peak value at 24 h in hepatopancreas of *M*. *nipponense* (Fig 9B). The observation in this study was in accordance with the previous report in *L*. *vannamei* and *M*. *nipponense* [18, 103]. The results indicated that the antioxidant defense system of *M*. *nipponense* was rapidly activated after being exposed to WSSV and had high efficiency to restore oxidative balance.

The interferon (IFN) response is the hallmark of antiviral immunity in vertebrates, characterized by induction of IFNs and the subsequent establishment of the cellular antiviral state. IFNs are a cluster of secreted cytokines with abilities to inhibit viral replication and modulate the function of immune cells [111]. The IFN regulatory factor (IRF) is a family of transcriptional factors that play critical roles in the activation of IFNs [112, 113]. IRF have markedly diverse roles in regulating gene expression network associate with immue system and development and response in immune cells. The first crustacean IRF gene was identified in *L*. *vannamei* by Li et al. [111]. In *L*. *vannamei*, IRF was up-regulated after WSSV infection, indicating that IRF could be activated in reponse to virus infection, and is a virus-inducible transcriptional factor [111]. The IRF in our transcriptome data was up-regulated. With WSSV infection, the mRNA expression of IRF in hepatopancreas of *M*. *nipponense* was significantly increased with a peak value at 48 h post-injection (Fig 9D). The results were accordance with previous studies in *L*. *vannamei* [111], indicating that IRF was involved in the reponse to WSSV infection and may play an important role in antiviral responses in *M*. *nipponensis*.

As a basic nutrient for all living organisms, iron was involved in many biological processes. Ferritin, an important iron storage protein in living cells, is vital for maintenance of iron homeostasis. Ferritin could converts ferrous to ferric through its ferroxidase activity, and then stores ferric as a mineral [114]. Ferritin was up-regulated in the hapatopancreases of prawns and shrimps challenged with WSSV [32, 115]. In *L*. *vannamei*, Ferrtitin was demonstrated that it can inhibiting WSSV replication [114]. The soma ferritin-like was up-regulated in our transcriptome data with the second highest up change (~10.02-fold) and different time-course WSSV challenge (Fig 9J). The results showed soma ferritin-like may play a role in the reponse against WSSV infection in *M*. *nipponense*.

## Conclusion

RNA-seq is a rapid and revolutionary technology for transcriptome analysis relative to traditional methods. In this study, we investigated the transcriptome profile of *M*. *nipponense* hepatopancreas when infected with WSSV using the Illumina HiSeq2005^TM^ technology. Abundant differentially expressed immune-related genes and signaling pathways were obtained. However, to further enhance our understanding about molecular interactions between *M*. *nipponense* and WSSV, further studies on the functionality of these genes will provide valuable information to find effective strategies to prevent viral disease. Besides, with whole genome sequence of this species is still unavailable, large number of transcripts obtained from this study could provide a strong basis for future genomic research on *M*. *nipponense*.

## Supporting information

S1 Fig The results of triplicated samples PCA analysis in control, moribund and survived groups. (TIF)

S2 Fig E-value distribution of the BLASTX matches of the transcriptome unigenes. This figure shows the E-value distribution of unigene BLASTX matches against the Nr protein database and the proportions.
(TIF)

S3 Fig Species distribution of the BLASTX matches of the transcriptome unigenes. This figure shows the species distribution of unigene BLASTX matches against the Nr protein database (cut-off value E < 10^−5^) and the proportions for each species.
(TIF)

S4 Fig Histogram presentation of KOG classification of 15,211 known protein annotated unigenes. Each bar represents the number of unigenes classified into each of the 25 KOG functional categories.
(TIF)

S5 Fig KEGG classification of *M*. *nipponense unigenes*. (all unigenes). (TIF)

S6 Fig Length distribution of the CDSs predicted by BLASTX and ESTScan. A means length distribution of the CDSs predicted by BLASTX and B means length distribution of the CDSs predicted by ESTScan.
(TIF)

S1 Table Differentially expressed genes (DEGs) in the comparison of control and moribund samples, control and survived samples. P-value was corrected for FDR. The absolute value of log_2_ (FPKM ratio in two compared groups) ≥ 1 and FDR ≤ 0.001 was set as threshold for the selection of significantly differential expressed genes.
(XLSX)
