# Supplementary figures and images for: A transcriptome study on Macrobrachium nipponense hepatopancreas experimentally challenged with white spot syndrome virus (WSSV)
Source: PLoS One. 2018 Jul 6;13(7):e0200222. doi: 10.1371/journal.pone.0200222 (PMC6034857; doi:10.1371/journal.pone.0200222)

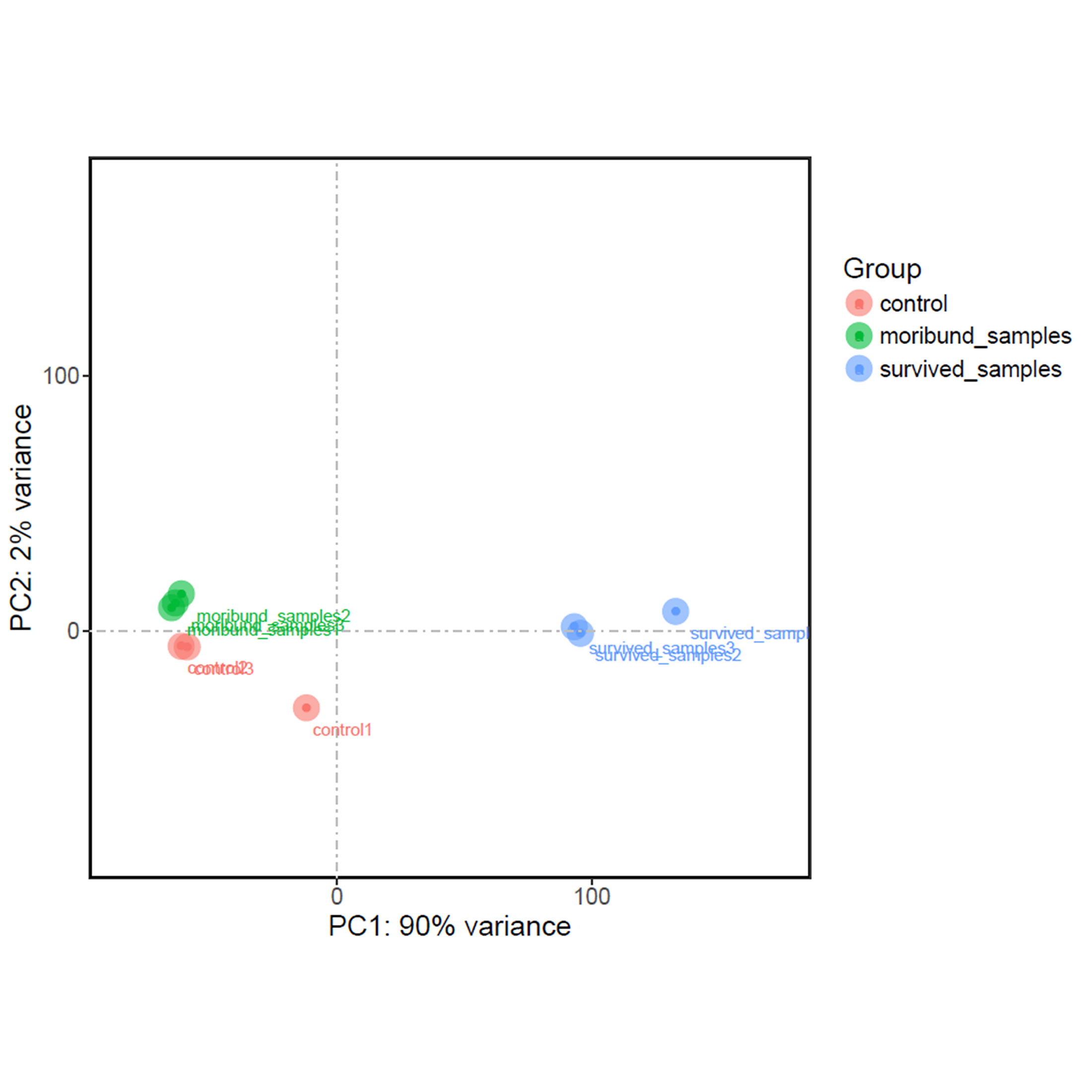

Supplement: S1 Fig — (TIF) [file pone.0200222.s001.tif]

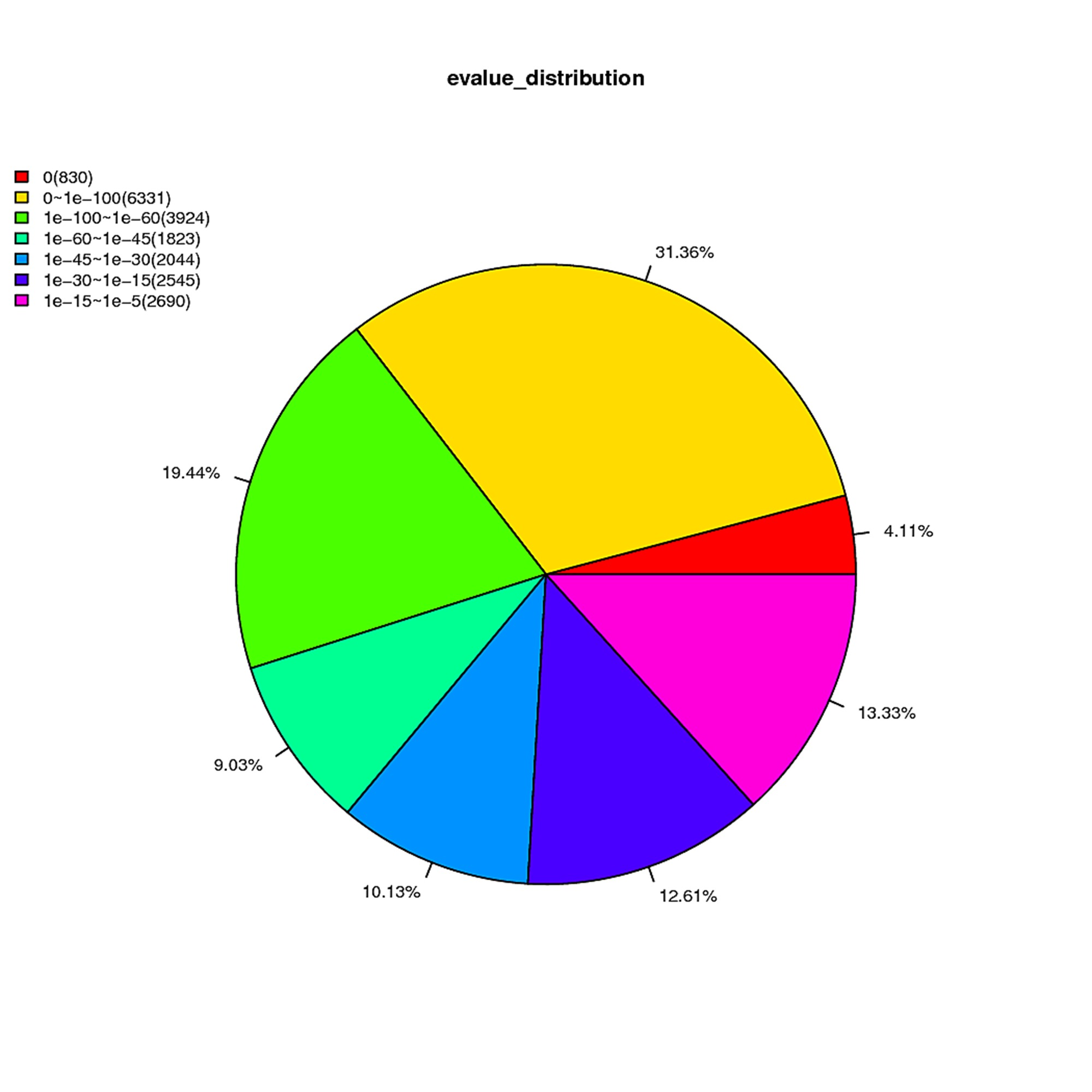

Supplement: S2 Fig — This figure shows the E-value distribution of unigene BLASTX matches against the Nr protein database and the proportions. (TIF) [file pone.0200222.s002.tif]

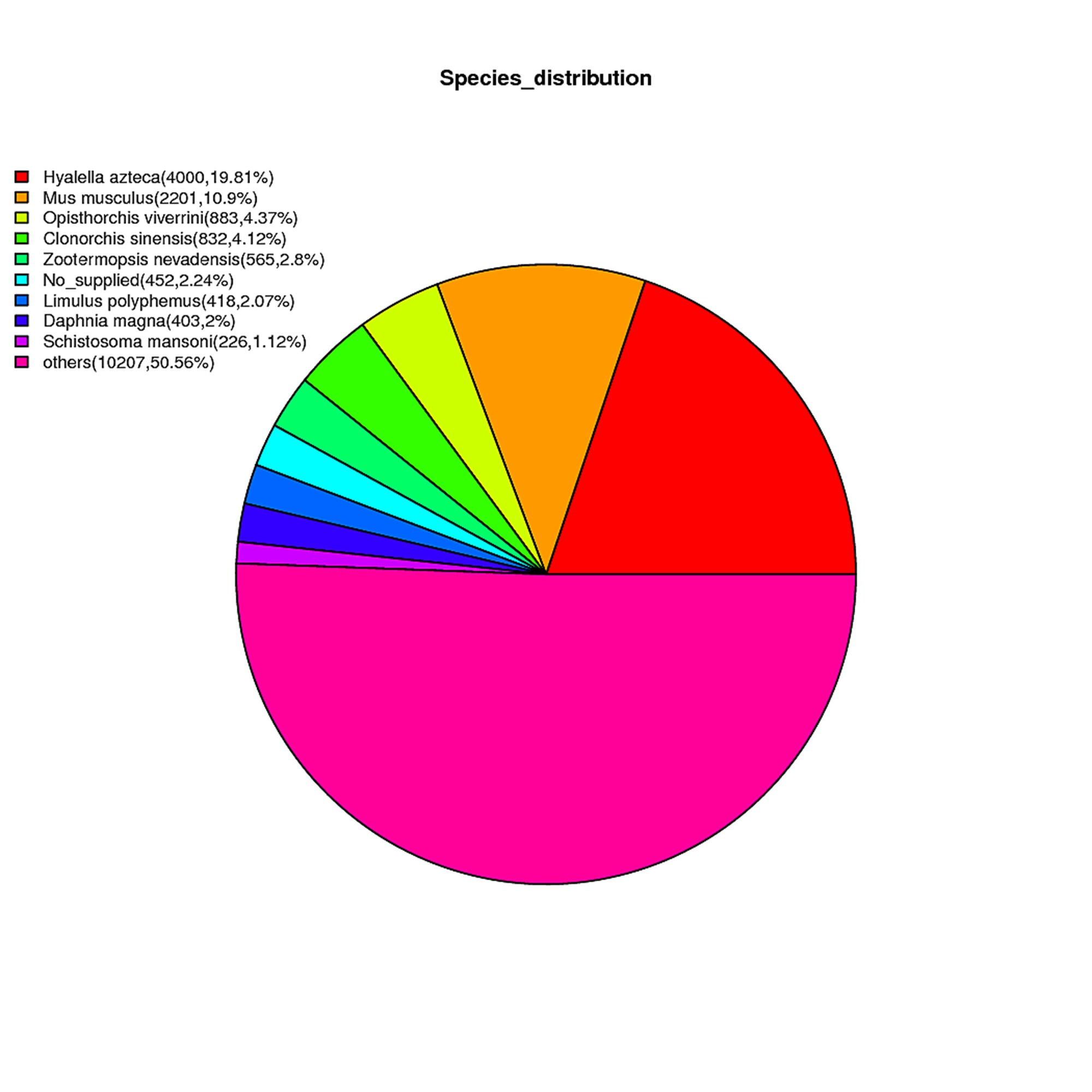

Supplement: S3 Fig — This figure shows the species distribution of unigene BLASTX matches against the Nr protein database (cut-off value E < 10−5) and the proportions for each species. (TIF) [file pone.0200222.s003.tif]

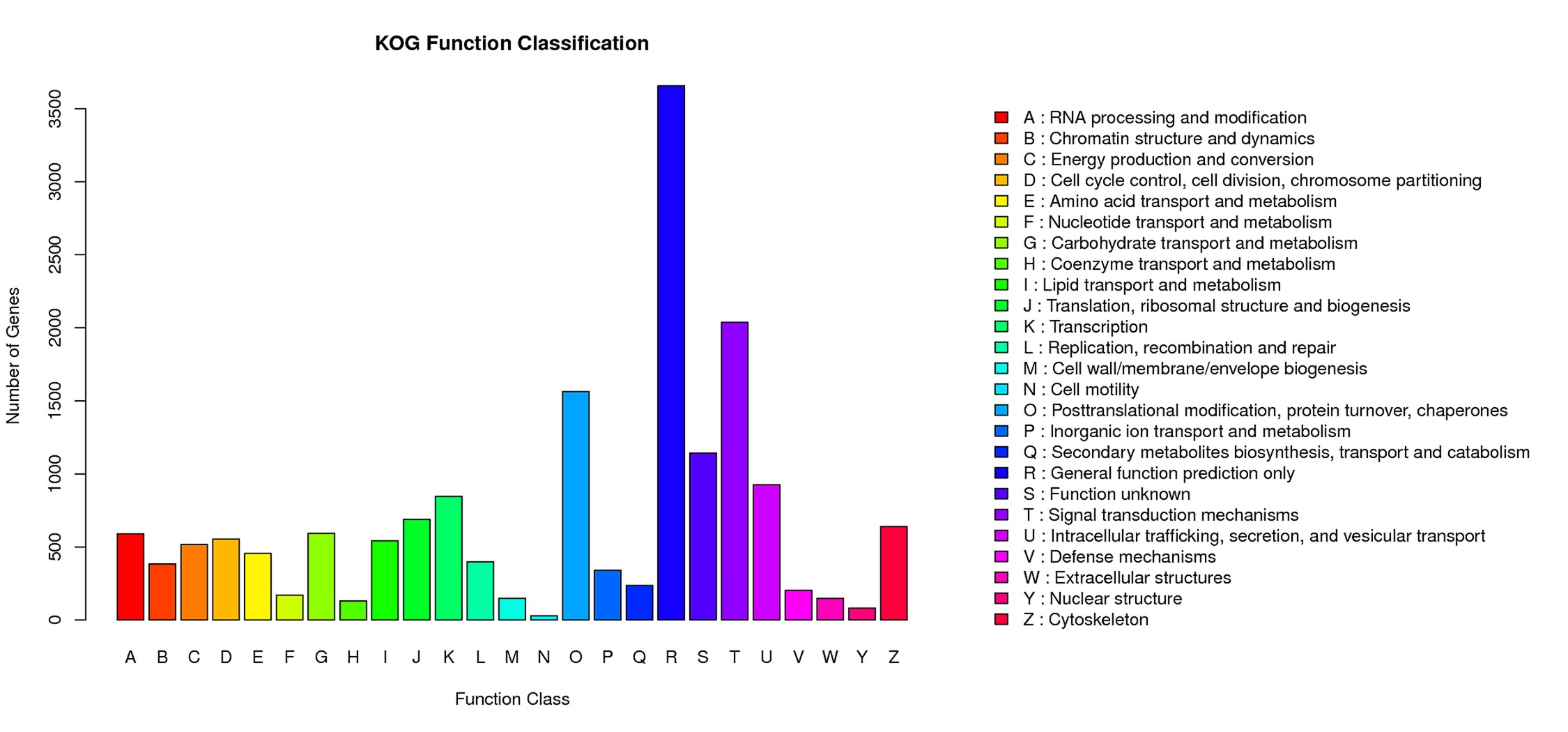

Supplement: S4 Fig — Each bar represents the number of unigenes classified into each of the 25 KOG functional categories. (TIF) [file pone.0200222.s004.tif]

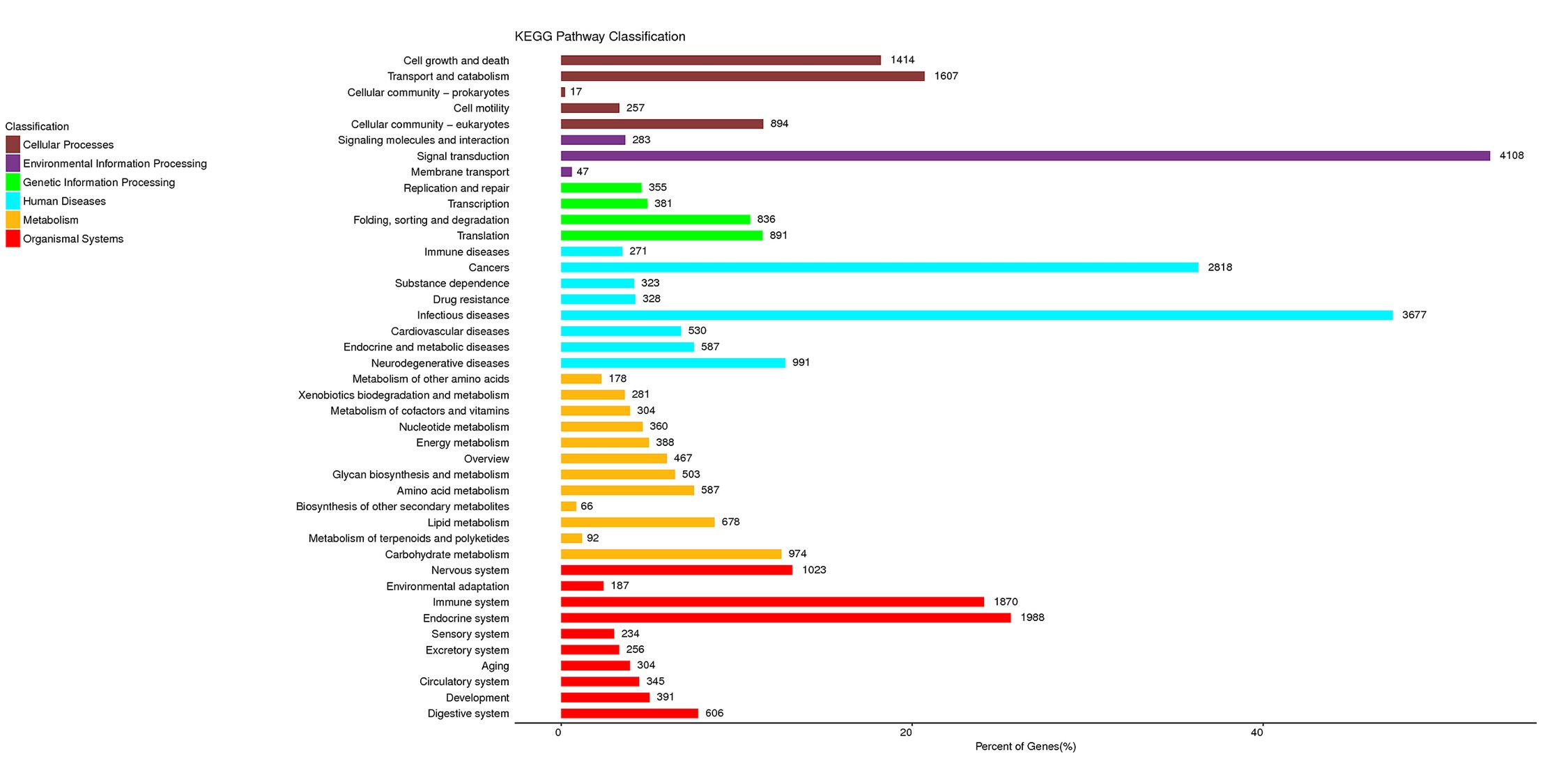

Supplement: S5 Fig — (TIF) [file pone.0200222.s005.tif]

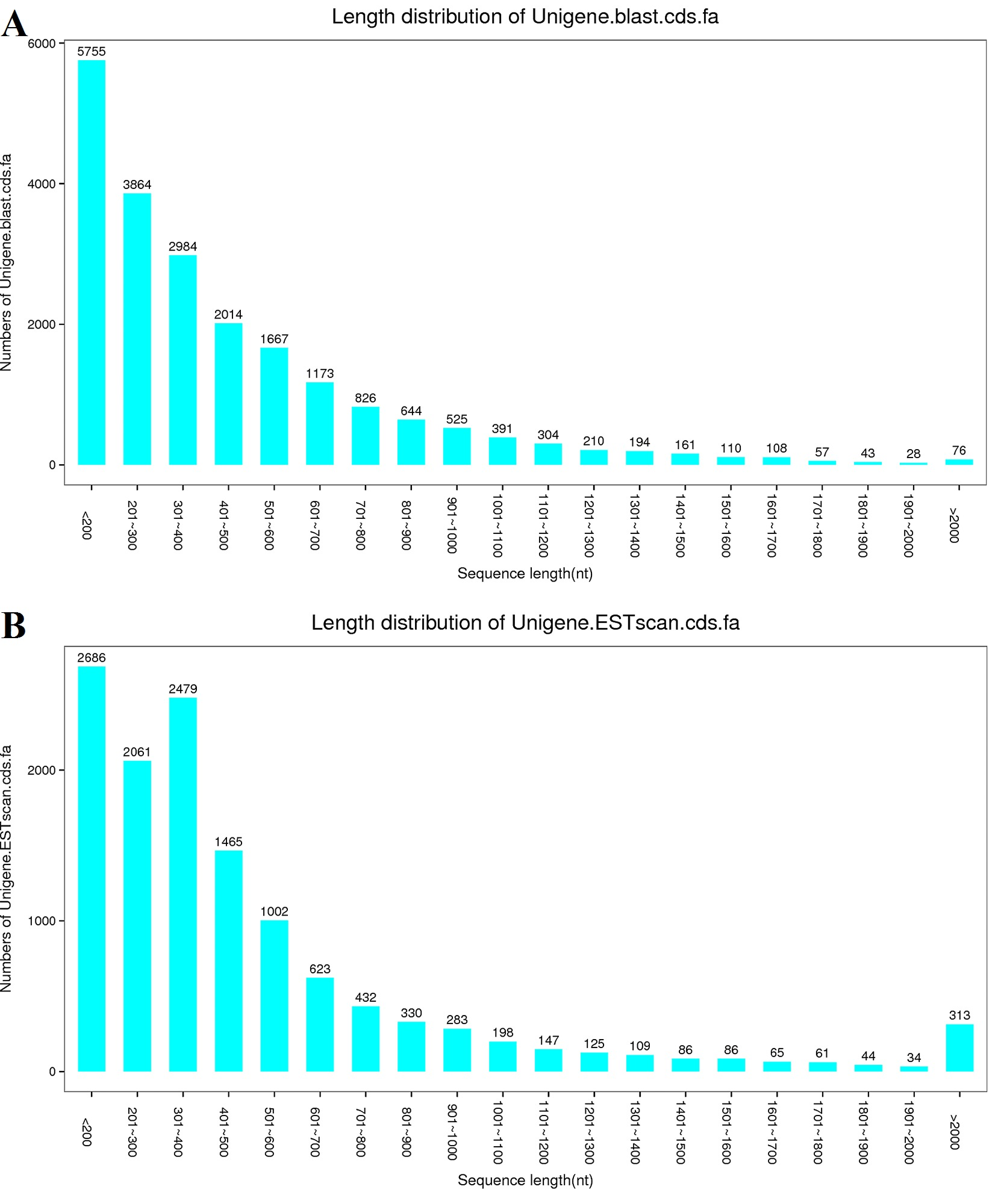

Supplement: S6 Fig — A means length distribution of the CDSs predicted by BLASTX and B means length distribution of the CDSs predicted by ESTScan. (TIF) [file pone.0200222.s006.tif]
